# Hop Compounds: Extraction Techniques, Chemical Analyses, Antioxidative, Antimicrobial, and Anticarcinogenic Effects

**DOI:** 10.3390/nu11020257

**Published:** 2019-01-24

**Authors:** Maša Knez Hrnčič, Eva Španinger, Iztok Jože Košir, Željko Knez, Urban Bren

**Affiliations:** 1 Laboratory of Separation Processes and Product Design, Faculty of Chemistry and Chemical Engineering, University of Maribor, Smetanova ulica 17, SI-2000 Maribor, Slovenia; masa.knez@um.si (M.K.H.); zeljko.knez@um.si (Ž.K.); 2 Laboratory of Physical Chemistry and Chemical Thermodynamics, Faculty of Chemistry and Chemical Engineering, University of Maribor, Smetanova ulica 17, SI-2000 Maribor, Slovenia; eva.spaninger@um.si; 3 Slovenian Institute of Hop Research and Brewing, Cesta Žalskega Tabora 2, SI-3310 Žalec, Slovenia; Iztok.Kosir@ihps.si

**Keywords:** hops, extraction, antioxidative effects, antimicrobial effects, anticarcinogenic effects

## Abstract

Hop plants comprise a variety of natural compounds greatly differing in their structure and properties. A wide range of methods have been developed for their isolation and chemical analysis, as well as for determining their antioxidative, antimicrobial, and antigenotoxic potentials. This contribution provides an overview of extraction and fractionation techniques of the most important hop compounds known for their health-promoting features. Although hops remain the principal ingredient for providing the taste, stability, and antimicrobial protection of beer, they have found applications in the pharmaceutical and other food industries as well. This review focuses on numerous health-promoting effects of hops raging from antioxidative, sedative, and anti-inflammatory potentials, over anticarcinogenic features to estrogenic activity. Therefore, hops should be exploited for the prevention and even healing of several prevalent diseases like cardiovascular disorders and various cancer types. New ideas for future studies on hops are finally presented: computational investigations of chemical reactivities of hop compounds, nanoencapsulation, and synergistic effects leading to a higher bioavailability of biologically active substances as well as the application of waste hop biomass from breweries for the production of high-added-value products in accordance with the biorefinery concept.

## 1. Introduction

Over the last decades, the scientific world has turned its focus on exploring the biological effects of plants used in folk/traditional medicine. Such interest seems justified as almost all described effects of healing plants were also demonstrated through several in vitro and even in vivo studies. Therefore, it is reasonable to continue exploring in this field as gained knowledge represents the basis for development of new food supplements for disease prevention or even for the design of novel drugs, especially for the most prevalent threatening diseases of our time such as cardiovascular disorders, diabetes, Alzheimer’s, and even cancer [1,2,3,4]

Increased demand for material goods is related to an increased occurrence of certain diseases that can be mostly ascribed to inappropriate dietary habits and to a stressful rapid way of life [5,6] The food industry is in its prime time; however, along with obvious advantages this also brings numerous harmful consequences. Plain, organically grown food is becoming a privilege of the wealthy. Whereas, the diet of the poor and the middle class increasingly consists of almost exclusively anti-nutritional aliments filled with synthetic sugars, sensory properties enhancers, growth hormones, and preservatives [7]. Listed substances are undoubtedly connected with the occurrence of several most prevalent diseases as cardiovascular or neurological disorders and cancer [7]. 

**Figure 1:**
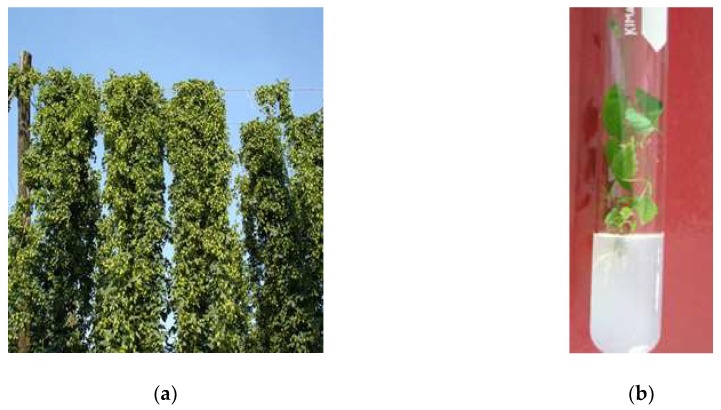
(**a**) Hop plant (*Humulus lupulus* L.) growing on a trellis. (**b**) Tissue cultured hop plant (*Humulus lupulus* L.). Photos were taken by Dr. Zala Kolenc at the premises of Slovenian Institute of Hop Research and Brewing.

Hop plant (*Humulus lupulus* L.), presented in Figure 1, has been in a continuous use for centuries or even a millennium mostly as an ingredient of beer, although some of its medicinal properties have been known as well. Nowadays its sedative effect is the most well-known, certain food supplements on its basis already exist for treating sleep disorders [8]. Several in vitro and in vivo studies also show that certain hop compounds carry a potential for becoming novel anticancer agents as they exert significant, numerous, and diverse beneficial biological activities. Is it therefore of utmost importance to pursue the investigation of in vivo potential of hop and hop constituents as novel drugs or anticancer agents.

Numerous publications on isolation of active compounds have appeared over the past two decades regarding a wide range of natural flavor formulations that are readily available including essential oils, herb and spice extracts, flavor substances fractionated from essential oils, or botanical extracts and natural flavor substances produced by fermentation [9]. The composition of these formulations depends on the nature of the solvent and on the applied extraction/isolation method. A considerable amount of effort has been invested in producing with hop extracts high flavonoid or polyphenol content. Polyphenols in hops have been studied for various reasons, especially due to their visible role in industrial applications as natural additives with antimicrobial and/or antioxidant properties. Technologies used to isolate compounds with a certain biological activity and to produce natural flavor formulations are well established, but several involve the use of conventional solvents like alcohols (methanol and ethanol), acetone, diethyl ether, or ethyl acetate and the use of methods that are energy intensive. Solvents that are produced from fossil fuels need to be tightly controlled with respect to their residual levels. The residues of the applied solvents may also remain in the final products bearing a detrimental effect on human health. This requires additional purification steps that are time-consuming and increase the total process cost [10]. In applying the ideologies of green chemistry, water and carbon dioxide clearly represent safer solvents and the use of a renewable feedstock possesses the widest acceptability.

The use of various gases in the sub- or supercritical state as extracting solvents has been under investigation for nearly half a century. A large number of natural products has been extracted with carbon dioxide and its commercial applications in the food industry are already well established. The earliest examples of such processes are decaffeination of coffee and production of hop extracts [11]. Development of such processes and their scaling up are still highly topical. Several modifications of these processes have been reported with respect to the extraction procedure, the choice of an appropriate solvent and cosolvent for the isolation of the desired compound and the tunability of operating conditions. New perspectives have also opened by the introduction of unconventional supercritical solvents, such as noble gases and their mixtures, SF_6_, and of course water as the cheapest solvent. However, supercritical CO_2_ still remains the solvent of choice for these operations and indeed more than 90% of supercritical fluid extractions have been performed with CO_2_ as the supercritical solvent, mainly because of its easy penetration inside plant materials and high solvent power. Disadvantageously, its use is largely limited to the processing of dry raw materials and compounds of low polarity as well as low molecular weight.

It this review article we collected the current knowledge on the methods for isolation, characterization, and determination of antioxidative, antimicrobial, and antigenotoxic potentials of compounds found in hops and combined it with the results of in vitro and in vivo studies on their biological effects. 

## 2. Methods of Isolation, Characterization, and Determination of Antioxidative, Antimicrobial, and Antigenotoxic Potentials of Main Chemical Compounds in Hops

### 2.1. Isolation of Main Chemical Compounds from Hops

Distillation has been traditionally used for obtaining essential oils from hops. Different approaches have been followed. The essential oil was obtained by passing steam through ground hops and removing the oil from the condensate by extraction with ether. The process took approximately 4 h for distillation of a 100 g sample of coarsely ground hops in 3 L of water. The effect of reducing the time taken for the usual steam distillation has been studied [12]. In 1969, US Patent 3436319 A was issued [13], by Freiherr Von Horst and Kellner, for their “Thin Layer Steam Distillation of Hop Oil Extract”. The process is described as continuous and was proposed for obtaining the essential oil of hop preparations. The main feature of this process was the advantage of completely recovering the oil of hop from the steam distillate and simultaneously producing a residual hop extract containing other components of said extract in substantially unaltered form.

Traditional isolation methods are more and more frequently replaced by the advanced techniques combining sample preparation, separation, detection, and identification. Application of liquid carbon dioxide (LCO_2_) in the range of temperatures from 20 up to 25 °C and in the pressure range from 50 to 60 bar, respectively, was for example reported in 1966 for the extraction of hops [11,14]. Stability of the obtained extract was however insufficient and has undergone a chemical change during storage.

An alternative approach was reported in 1977 by Laws and coworkers [15]. Extraction of hops with supercritical CO_2_ was performed at temperatures between 40 and 50 °C and pressures between 150 and 400 bar. A high-quality hop extract was also obtained by using LCO_2_ at relatively low temperatures. A patent of Muller [16] described the extraction at a temperature below the critical temperature (approximately 31 °C) but at a pressure higher than the critical one (73.25 bar). Next variation included the addition of a small amount of ethanol as a solvent to the liquid carbon dioxide [17]. The choice of an appropriate solvent represents the key factor for isolation of the compound of interest and depends on a number of factors, including dissolving power, selectivity, inflammability, volatility, and cost. Selectivity of LCO_2_ is for example used to provide hop extracts free of hard resins and polyphenolic materials which is not the primary focus of this review article that mainly covers extraction and separation methods for isolation of acidic compounds contained in hop flowers and their antitumor, antimicrobial, and antioxidative effects. CO_2_ is thus a substitute for organic solvents of a highly nonpolar character, such as hexane and pentane. The range of organic species which dissolve in LCO_2_ includes hop resins and aroma components, paraffins, naphthenes, olefins, alcohols, aldehydes, phenols, esters, carboxylic acids, amines and nitrogen heterocycles, aromates, ketones, ethers, amides, and nitrites [11], but not polyphenols.

Ethanol as a polar solvent dissolves a broader range of hop components and represents a suitable solvent for extraction of polyphenols, compounds with structural phenolic features, which can be associated with different organic acids and carbohydrates. Typically 90% ethanol is used and the resulting extract possesses a very similar composition to the original hops. However, the total content of polyphenols is still lower with respect to the other groups of substances [9]. Besides ethanol, scientific literature reports alternative organic solvents—such as methanol, acetone, and ethyl acetate—as effective extractive media for polyphenols as well as, to a lesser extent, propanol, dimethylformamide, and their combinations [18]. Considering industrial applications, the use of organic solvents is limited due to the fact that nontoxic solvents and the maximum residue levels of several solvents in the extracted foodstuff are strictly specified. Consequently, for the manufacture of hop extracts by breweries, carbon dioxide still remains the most applied solvent. High pressure or supercritical fluid extraction is especially suitable for isolation/fractionation of valuable ingredients from natural raw materials with limited solubility in CO_2_ at moderate pressures. The extracts contain nearly all essential oils in hops, as well as a sufficiently high ratio of α-acids (humulones) and less bitter lupulones, besides other components such as hard resins and traces of triglycerides, waxes, chlorophylls, and inorganic salts [19]. The main feature distinguishing CO_2_ extracts from those prepared with conventional extraction is the lack of traces of undesired organic solvents. Therefore, supercritical CO_2_ extraction has become the industrial process of choice for the production of brewery ingredients. 

Despite the abundant literature on extraction solvents and techniques for polyphenols from a different plant and herbal sources, there is surprisingly little information available on the effect of extraction conditions on the polyphenol content and antioxidative activities of hops and its products. Namely, the solubility of a solute depends on the solvent density that may vary considerably with changing extraction conditions, especially when operating in the sub- or supercritical region. With a higher temperature, moving towards the critical point, the solvent power increases. Optimum conditions for the extraction of a particular solute has to be therefore established experimentally by determining phase equilibria of this substance in a given solvent.

Numerous studies have shown that organic solvents without the addition of water represent poor solvents for the extraction of polyphenols [20]. Methanol or ethanol can also be mixed with water in different ratios. Alcohol concentration thus plays a key role in polyphenol recovery [20].

Despite several disadvantages, liquid–liquid and solid–liquid extractions are still the most commonly used isolation procedures. For several years, conventional techniques have been widely accepted, mainly because of their ease of use, efficiency, and wide-ranging applicability [21]. Typically, sterilized contaminant free components should be obtained from food and natural tissues in their chemically natural state. Harmful components from nutraceutical products should be removed; heavy metal recovery and enantiomeric resolution are also possible. Currently, solid phase microextraction is successfully applied for the characterization of aromatic properties of hops and other plant raw materials. On the contrary, for the isolation of nonvolatile compounds, solid-phase extraction, and solvent extraction are successfully used [22]. For sample preparation, including purification and/or isolation of polyphenolic compounds, solid-phase extraction in offline columns has also become a popular and effective method. Moreover, accelerated solvent extraction has been recently applied for the extraction of bitter acids from hops and hop products. Finally, low-temperature levels, high yields, and a short process time represent the main advantages of ultrasound-assisted extraction. The procedure usually requires additional cooling, the main part of the applied energy is thereby transferred into heat to protect heat-sensitive substances. Due to cavitation, the cells of the plant material are highly disrupted [23].

Supercritical fluid extraction, routinely used for the production of bitter acid extracts for the beer brewing industry, represents an effective method for the isolation of both volatile and nonvolatile compounds of hops including essential oils. Operating conditions, temperature, and pressure used for the extraction markedly affect the composition of the obtained extract. Relatively high recovery of volatile compounds is obtained at lower temperatures, whilst elevated pressures and temperatures favor high recovery of bitter acids and resinous compounds. Unfortunately, under the conditions used in the extraction with supercritical carbon dioxide for the preparation of hop extracts for the brewing industry, a large group of biologically active prenylflavonoids (prenylchalcones and prenylflavanones) remains in the plant material. Besides CO_2_, water is also relatively often used as an extraction medium, mainly due to the ability to become an excellent solvent for organic compounds and a very poor solvent for inorganic salts above its critical point (374 °C, 218 atm). This means that the same solvent can be used to extract the inorganic and the organic components, respectively. Products obtained in this way are solvent-free, without the presence of coproducts and the operational temperature is in the case of CO_2_ low [24]. The application of supercritical fluids (SCF) for extraction of natural substances at even higher pressures (over 70 MPa) than in conventional SCF extractions gives rise to new products from known plant materials such as the isolation of less soluble substances. A method for extraction and dissolution of hop acids, including α-acids, iso-α-acids, β-acids, and their derivatives in aqueous media, also comprising of the formation of quaternary ammonium salts of hop acids with quaternary ammonium compounds or mixtures thereof has been recently developed by Mertens and Pascal [25]. Hop acids containing matter is mixed with one or more quaternary ammonium compounds. Quaternary ammonium salts of hop acids are thereby formed. Their specific advantage is a high solubility in (acidic) aqueous medium compared to the corresponding hop acids in the free acid form. This invention further relates to the use of quaternary ammonium salts of hop acids in the beer brewing process and represents an effective concept of improving the utilization of hop acids, including α-acids and bitter taste contributing iso-α-acids, in this process. 

### 2.2. Analytical Methods

High resolution and the ability to provide precise and accurate qualitative and quantitative data put forth gas chromatography coupled with mass spectrometry (GC–MS) analyses as a valuable tool for taxonomic studies of plants. Various phenolic compounds have been found in beer using different detectors, such as coulometric, electrochemical, and photodiode arrays; ultraviolet–visible spectrophotometry; and low-resolution mass spectrometry [26]. More than two hundred compounds such as essential oils, prenylflavonoids, and bitter acids usually classified as α-acids and β-acid, can be detected and further separated. In one single run, their quantities can be estimated by the means of capillary GC analysis.

Main components of hop essential oils such as monoterpenes and sesquiterpenes comprising humulene, bisabolene, caryophyllene, farnesene, and elemene skeletons are commonly determined by the evaluation of the total volatile content by GC–MS [22]. The method requires the determination of retention parameters (t_R_, calculated retention index and Kovats retention index) and m/z values of molecular ions for selected compounds from hop essential oils [22]. The main advantage of the method is that essential oils obtained by steam distillation are ready to use for subsolvent GC analysis after appropriate dilution without additional purification.

α-, β-, and iso-α-acids are known for their high potential to oxidize. Development of simple preparative methods (which exclude other hop constituents such as polyphenols, lipids, waxes, and polysaccharides) for the bitter acid oxide fractions is essential due to their effect on beer properties as well as their potential health benefits [27]. A total content of matured hop bitter acids (MHBA), primarily composed of α-acid-derived oxides, is determined by a quantitative analytical method using high-performance liquid chromatography (HPLC) frequently coupled with atmospheric pressure ionization tandem mass spectrometry (APCI-MS-MS) or with negative electrospray ionization mass spectrometry. “General, HPLC analyses have unfortunately displayed a lack of reproducibility, the disadvantage of consumption of organic solvents and only the major compounds, i.e., the humulones and iso-humulones can be examined by this methodology.” Xanthohumol has been determined in hops by HPLC using UV detection. Though, this technique lacks deficient sensitivity and for the quantitative determination of the minor prenylflavonoids. Higher sensitivity is enabled by tandem mass spectrometry (MS-MS). Liquid chromatography (LC) coupled with (tandem) mass spectrometry has been successfully applied to the quantitative analysis of prenylflavonoids [28].

A variety of phenolic compounds, such as hexosides, dihexosides, pentosides, and quinic conjugates, such as feruloyl quinic acid, caffeic acid-O-hexoside, coumaric acid-O-hexoside, sinapic acid-O- hexoside, catechin-O-dihexoside, kaempferol-O-hexoside, and apigenin-C-hexosidepentoside, in beer extracts, of which some have been reported for the first time in beer, were efficiently determined by a LTQ-Orbitrap high resolution mass spectrometer [29]. However, techniques employing high-resolution mass spectrometry are still under intense investigation, since data on identification of the phenolic profile by high-resolution mass spectrometry in analysis of hops compounds are still relatively scarce. These techniques, including ion trap quadrupole-Orbitrap-mass spectrometry (LTQ-Orbitrap-MS), which provides single-stage mass analysis that supplies molecular weight information, as well as two-stage mass analysis (MS/MS) and multi-stage mass analysis (MSn) that provide structural information, have been demonstrated as a reliable tool for the structural elucidation of unknown compounds in complex mixtures such as the total hop extracts. 

Isoxanthohumol can be metabolized in the human liver to form 8-prenylnaringenin. Prenylnaringenin is an isomerization product of desmethylxanthohumol and until now, known as the most potent phytoestrogen isolated [30]. Specific cytochrome P450 enzymes are responsible for the O-demethylation reaction. The enzymes that convert isoxanthohumol and 8-prenylnaringenin to their most abundant metabolites were identified. 

Evaluation of the results obtained by chemometric methods provides data on phytochemical composition, which is crucial for the standardization and quality control of plant raw materials required by food or pharmaceutical industry.

### 2.3. Methods and Techniques for Determination of Antioxidative Activity

Polyphenols are extremely important for the physical stability of beer during storage [31]. Oxidation and polymerization of endogenous polyphenols and their interaction with proteins represent the main reason for beer turbidity. Catechin and proanthocyanidins (dimers and trimers of catechin, epicatechin, and gallocatechin) have displayed haze-forming activity with peptides in model systems [32].

Total phenolics and antioxidative activities of hop extracts are commonly determined by well-established methods. 2,2-diphenyl-1-picrylhydrazyl radical (DPPH*) scavenging assay is used to evaluate the antioxidative activities of the extracts, whilst the total phenolics are determined by the reduction of phosphotungstic acid and phosphomolybdic acid [33]. Besides, other in vitro antioxidant assays, such as the oxygen radical absorbance capacity (ORAC) assay, the hydrogen peroxide scavenging (HPS) assay, and the linoleic acid (LA) assay, have also been reported for evaluation of oxidative stability. Using the ORAC assay, cinnamic, caffeic, and ferulic acids; gallocatechin, xanthohumol; and myricetin were described as the most active in hydroxyl and peroxyl radical-scavenging [32]. The effect of phenolic compounds was related with vitamin C, using ABTS and DPPH-scavenging assays. Antioxidant activities are expressed as vitamin C equivalent antioxidant capacity (VCEAC). VCEAC results for phenolic compounds were gallic acid > quercetin > epicatechin > catechin > vitamin C > rutin. It was proposed that monomeric and oligomeric flavonols have the ability to limit LDL oxidation. It was demonstrated that among phenolic compounds, flavonoids with a prenyl group are more effective as inhibitors of LDL oxidation [31,33].

Most of the studies were focused on the antioxidant activity of literature-known compound classes in beer. There is a lack of studies on identification of the key antioxidants in beer. For that purpose, the target antioxidants should be isolated, purified, and determined in their chemical structure by the means of LC-MS and 1D/2D NMR [34].

### 2.4. Methods and Techniques for Determination of Antimicrobial Potential

Hops have a long history of use as a natural preservative in beer due to high concentrations of unique bitter acids that inhibit the growth of Gram positive bacteria already at surprisingly low concentrations. *Staphylococcus aureus* is one of the most common Gram positive bacteria causing food poisoning. Its source is not the food itself, but the humans who contaminate the food after it has been processed. Despite the well-known high antibacterial, antifungal, and antiviral activities of hops, there is a lack of information about antimicrobial potentials of individual hop compounds. However, lupulone, humulone, isohumulone, and humulinic acid have shown high antimicrobial activity against certain bacteria like *Bacillus subtilis* 168 [35]. Isolation of such individual chemical compounds and their subsequent analyses are quite tricky and time-consuming, especially in the case of a complex biological matrix such as the hop extract [36]. Recent investigation reports that seven flavonoids, among them two natural (α,β-dihydro xanthohumol and 8-prenylnaringenin) showed significant activity against methicillin sensitive and resistant *Staphylococcus aureus* and *Staphylococcus epidermidis* [37]. *Aspergillus niger* is a clearly visible spoiling agent of bakery goods that forms black-centered spots on the surface of products. A typical opportunist, *Candida albicans* is the microbe responsible for most clinical yeast infections, e.g., in mouth infections [38].

There is an urgent need for new antimicrobial ingredients specifically targeting oral pathogens such as *Listeria monocytogenes*, *Bacillus cereus*, *Staphylococcus aureus*, and *Lactobacillus* sp. Despite the availability of robust preservatives food spoilage and poisoning caused by microorganisms remains a problem that has not yet been brought under adequate control. Natural preservatives as an alternative to the artificial ones are therefore needed to achieve sufficiently long shelf-life of foods and a high degree of safety with respect to foodborne pathogenic microorganisms. Several studies have been performed to investigate whether classical hop extracts, supercritical hop extracts or extracts obtained by isomerization of supercritical hop extracts, could be used as antimicrobial agents against foodborne pathogens and also against microorganisms capable of causing food spoilage due to the high content of phenolic phytoalexins, e.g., flavonoids. Screening of plant extracts and their constituents has a well-established history for searching novel anti-infective agents effective against cariogenic and periodontal bacteria [39]. Nowadays, plant antiseptics are incorporated into a whole range of oral products. The substances responsible for this activity are mainly the hop acids, which are classified as α-acids (humulones), β-acids (lupulones), and their oxidation products. These substances consist of a mixture of homologous compounds [20]. Residues attained in the process of hops extraction, which comprises a waste product from the brewing industry, may be used as a cheap source of natural compounds with dual functions (as an antioxidant and antimicrobial agent) of wide application.

Different techniques according to the Standard Test method ASTM E 2149-01 have been reported to assess antimicrobial activity of Immobilized Antimicrobial Agents Under Dynamic Contact conditions (E2149-01, 2002) [40,41,42,43].

The most extensively used techniques to investigate the antibacterial activity of natural substances and plant extracts are diffusion methods which are based on the use of discs or holes as reservoirs containing solutions of substances to be examined according to Brantner and Grein [44], which studied plants of 28 families, selected on the basis of medicinal folklore reports and literature data, in a screening program. Aqueous extracts of plants used externally for treatment of infected skin lesions were tested for their antibacterial potential. The results indicated that ~60% of the plant extracts tested exhibited some level of antibacterial action. Hole-plate diffusion method is used in the case of pure compounds, whilst the cylinder diffusion method is used for the extracts.

However, in the case of solutions with low activity a larger concentration or volume is needed, which may be problematic when determining the antibacterial activity of extracts, including hop extracts. The limited capacity of discs means that holes or cylinders are preferably used. Among several phenolic compounds present in plant extracts, flavone, quercetin, and naringenin have been proved as highly effective in inhibiting the growth of microorganisms due to their interaction with nucleic acid or proteins [45]. Nonetheless, plant extracts generally contain a variety of flavonoids. The broad range of diverse chemical structures may reflect in different biological activities of extracts [46]. Indeed, diverse extraction procedures give extracts with characteristic phenolic profile and different proportion of single phenolic compounds and this certainly influences the antioxidative properties of the extracts.

Hop extracts obtained by supercritical CO_2_ extraction, show significant antibacterial potential against investigated bacterial strains. Xanthohumol has been proven to possess the highest activity against all tested strains. According to the literature, α- and β-acids, humulones and lupulones, and the isomerized forms of humulones might be considered as antimicrobial agents in hop extracts as well. Possible mechanisms of antibacterial activity of bitter acids and their derivatives might include the induced leakage of the bacterial membrane due to their highly hydrophobic character, especially of lupulone [47]. Among organic solvents used for Soxhlet extraction, methanol and ethanol have been proven as efficient solvents for isolation of compounds with high antimicrobial activity. On the other hand, when n-hexane is used as a solvent, the resulting extract is less effective than methanol and ethanol extracts against tested bacteria and fungi.

### 2.5. Methods for Determination of (Anti)Genotoxic Potential

Even though naturally occurring substances as an essential part of edible plants and their products should pose no health risk, we cannot presume that the same applies for isolated compounds in higher doses and for other formulations especially in the form of phytopharmaceutical drugs. Therefore, also in the case of a naturally occurring substance with proven health-promoting effects, testing for potential toxicity and use of limited concentrations is essential for safe use. Several testing methods and protocols exist for the toxicological evaluation of compounds, some even in silico [48].

Among possible toxic effects, genotoxic effects, causing a detrimental impact on the genetic material (DNA) tend to be in the center of scientific attention as they represent the leading cause of several prevailing diseases of our time, especially various cancer types. In order to prevent the formation of cancer and possibly other genetic diseases, one must try to avoid any exposure to genotoxic agents or/and include or increase the intake of chemoprotective substances capable of delaying, preventing, or inhibiting one or more cancer stages [49,50]. Various potential anticarcinogenic substances can be found also in hops and their effects are presented in the following chapter. 

The methods and tests for determining the genotoxic potential can, with minor modifications, in most cases be used for the determination of the antigenotoxic potential as well. The most widespread used genotoxicity test remains the Comet assay as it is straightforward, economical, and can be applied in numerous ways on various cells and tissues for investigating DNA damage at physiological conditions and concentrations [51]. As it is utilized for determining DNA damage and its repair, it is popular in human biomonitoring studies along with environmental studies and in assessing the occupational exposure to genotoxic agents associated with various diseases, and the intrinsic factors that affect DNA damage levels in humans. On the other hand, genoprotection by dietary and other factors as well as the exploration of the effect of repair gene polymorphisms can be addressed too [51,52]. The essence of the Comet assay is in that the studied cells are embedded in agarose, lysed, and electrophoresed at high pH, the DNA strands containing breaks thereby move towards the anode, which under fluorescence microscopy looks like a comet tail. The assay has received numerous improvements, especially by including various DNA modifications like oxidized bases and DNA alkylation with specific enzymes in addition to strand breaks that are primarily/easily detected/determined by the Comet assay. Among the most commonly used enzymes one can find endonuclease III, formamidopyrimidine DNA glycosylase (FGP), and 3-methyladenine DNA glycosylase II [51,53]. As already mentioned, the Comet assay additionally enables the determination of DNA repair, not only DNA damage. For determining DNA repair, DNA damage on cells is initially induced via ionizing radiation, UVC radiation, or other genotoxic agents and the chromosomal aberrations or micronucleus frequency is measured as it represents unrepaired DNA [54]. Then, a decrease of the DNA breaks (relative tail fluorescence) is monitored with the incubation time [52]. By introducing lesion-specific endonucleases after the lysis of the cells it is possible to observe the removal of specific base damages like oxidized and alkylated bases removed by the base excision repair and UV-induced cyclobutane pyrimidine dimers removed by the nucleotide excision repair [54]. The latter additionally enables to assess the repair capacity by measuring the increase of repair intermediates instead of the removal of the damage [54]. Therefore, the associations of investigators of the majority of diseases share a common belief that the Comet assay exhibits an enormous potential for the use in everyday clinical practice [52].

The Comet assay possesses several advantages over alternative methods such as using substrates with a low density of lesions and better simulating real-life conditions compared to other in vitro assays [54]. Only the quantitative polymerase-chain-reaction (PCR) method requires equally small amounts of DNA as the Comet assay; however, the limit of detection of the latter is at least an order of magnitude higher and therefore, inappropriate for a biomarker assay [54]. Compared to HPLC methods, the Comet assay is less precise, but better when estimating background levels, and its FPG-based modifications are even more accurate than HPLC [51]. 

Another universally applicable method for genotoxicity tests is the cytokinesis-block micronucleus (CBMN) assay. This method is used for determining the standard micronucleus index (MNi), representing a small nucleus lacking part(s) of a chromosome or lagging behind in anaphase during nuclear division [55,56]. This method can additionally be used also for measuring the nucleoplasmic bridges (NPBs), nuclear buds (NBUDs), cell death (necrosis or apoptosis), or nuclear division rate [53]. Therefore, the CBMN method remains widely applicable for determining chromosomal instability, cell death, and cytostasis through various direct and/or indirect measurements of several aspects of cellular and nuclear dysfunctions. An important advantage of the CBMN method lies in specifically restricted scoring to once-divided cells [55]. 

Numerous natural compounds exhibit phytopharmaceutical potentials. Therefore, to avoid unnecessary laboratory testing and to decrease research costs, it is reasonable to first perform the in silico analysis in order to reduce the number of potential candidates to be experimentally tested or to find novel, stronger candidates for laboratory tests. Even the European Food Safety Authority (EFSA) has proposed a computational approach to supplement the toxicity profiles of compounds [48]. There exist various computational approaches for investigating genotoxicity of specific compounds. One approach is to use the tools that search the existing (toxicological) databases and compare the substances on the basis of their structural elements or other properties with various mathematical/statistical operations [48]. A completely different approach that is also used by our research group is to perform computer simulations of chemical reactions of DNA with potential (anti)genotoxic agents on the basis of quantum mechanics and/or molecular dynamics. More insights are presented in the concluding chapter.

## 3. Main Chemical Compounds of Hops and Their Biological Effects

Hop plant was long recognized only for its sedative (for insomnia) and antimicrobial (beer-stabilizing) properties [57]. More concise studies revealed that hop plant or constituting substances possess several other biological properties such as strong antioxidative action, estrogenic activity, anti-inflammatory action, and several anticarcinogenic features like apoptosis-inducing, antimetastatic, antiproliferative, anti-invasive, or antiangiogenic properties [8,58]. The above-listed features of hop plants have been generally ascribed to the biologically active compounds belonging to the group of secondary (hop) plant metabolites. Their primary role is to protect the plant from the predators, parasites, extreme weather conditions, and other threats [24]. 

A larger part of our diet consists of plants and their products. Therefore, exploring and exploiting plants with significant healing capacities should remain the main focus of scientific research as it has numerous advantages. Compounds from plants can be easily administered (added) to the diet as they exert low or are even devoid of harmful side effects. In addition, synergistic effects can importantly enhance their action or also action of existing drugs and therapies [24,59]. Moreover, such plants and their products, including extracts, can be exploited for prevention, reversing the progress or even healing of various widespread diseases. Finally, secondary plant metabolites are characterized by specific responses depending on the environment [24]. The same compound can, therefore, have antioxidative and proliferative effects in a healthy living cell and a pro-oxidative effect in a tumorigenic cell causing the induction of apoptosis [24]. 

According to the genetic profile, three significantly different groups of hops exist the North American, Asian, and European hops. Within these groups, numerous distinctive hop cultivars are found, as a consequence of the quest for better beer aroma [6,57].

**Figure 2:**
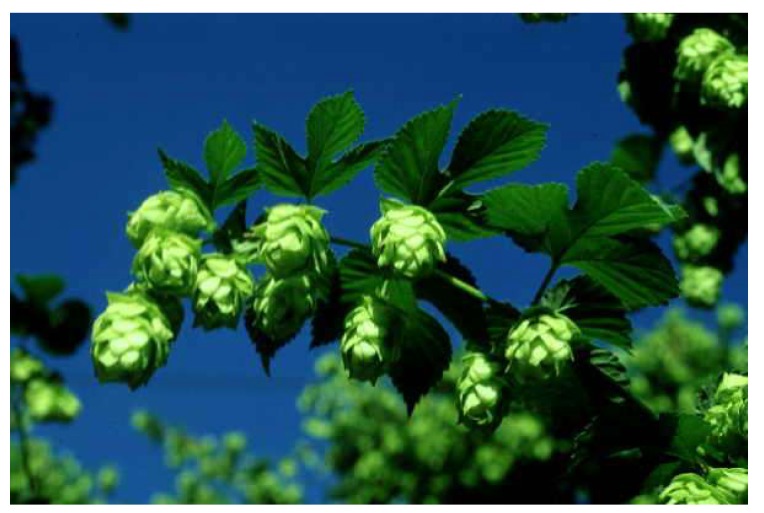
Hop cones of the female hop plant (*Humulus lupulus* L.). The photo was taken by Prof. Dr. Iztok Jože Košir at the premises of Slovenian Institute of Hop Research and Brewing.

**Figure 3:**
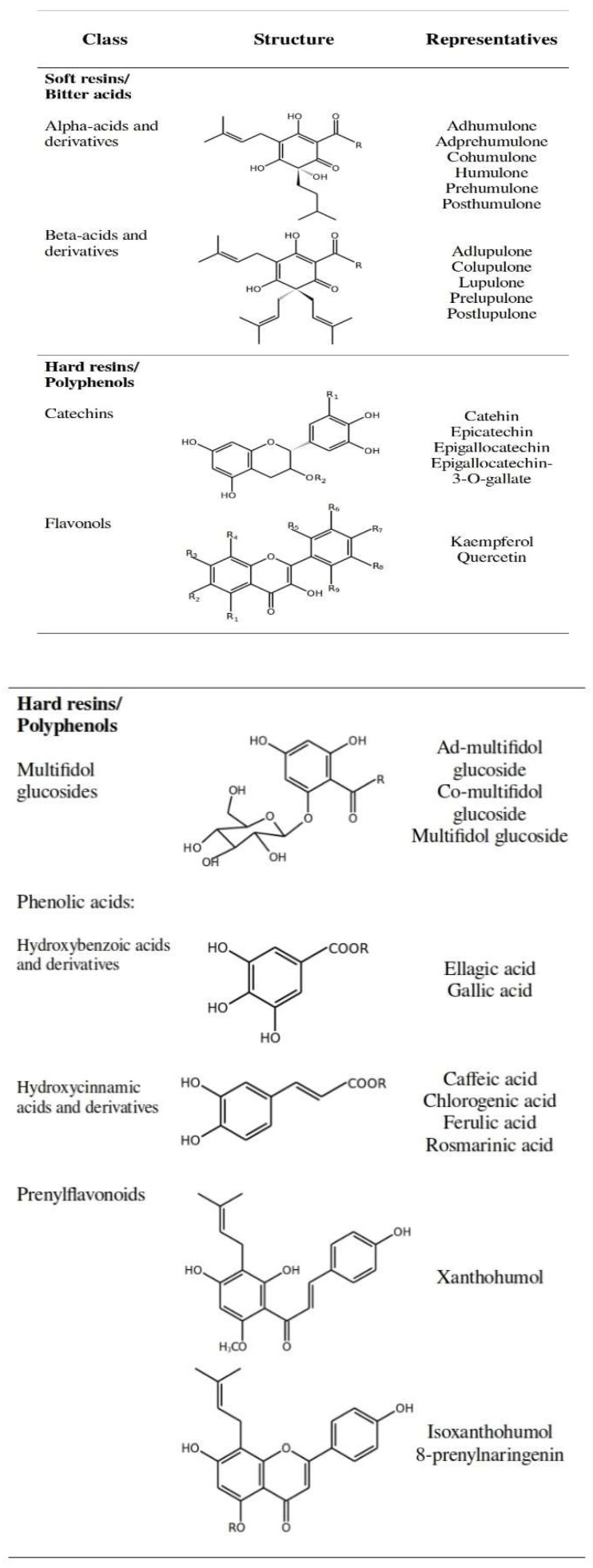

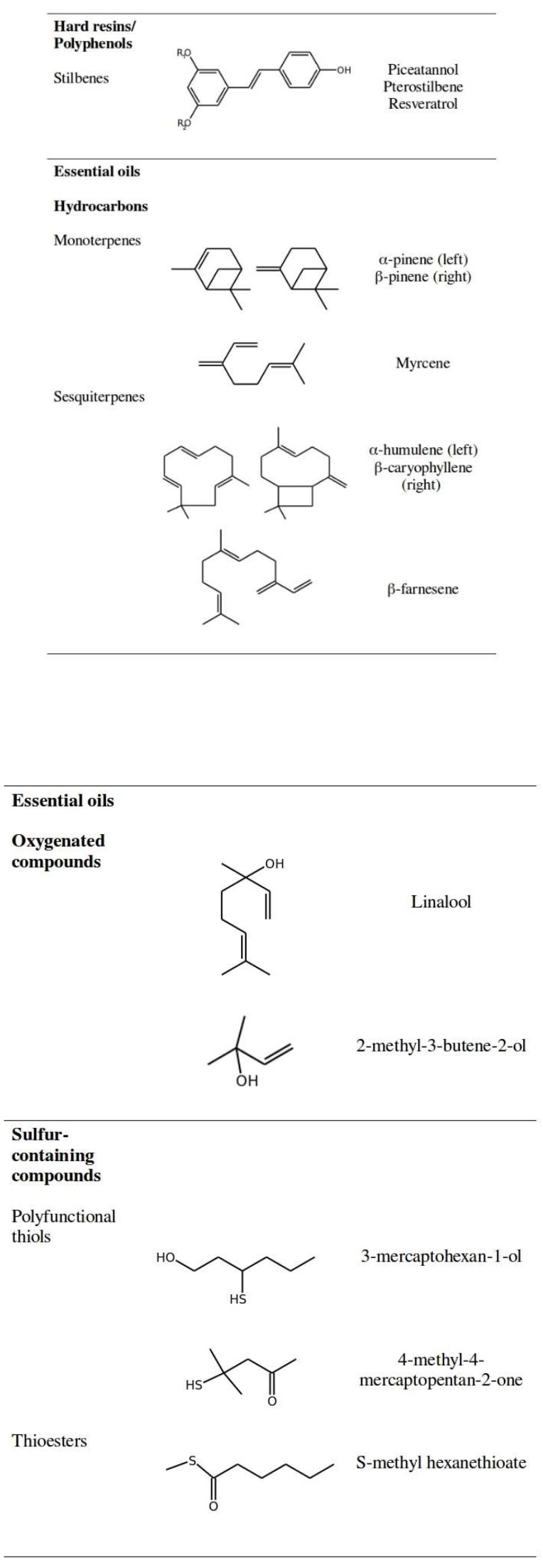
The main groups of compounds found in hops, their (skeleton) chemical structures, and some typical representatives. Chemical structures of all typical representatives of soft and hard resins are collected in supplementary material (Figure S1).

Hop cones of the female hop plant, presented in Figure 2, are in the center of the scientific attention as an exclusive part of the hop plant used for the production of beer owing to their bitter taste, aroma and antimicrobial properties [60]. Hop cones contain several functional groups of compounds; the non-nutrient portion (secondary metabolites) can be divided into three major classes: bitter acids and their derivatives, polyphenols, and essential oil components [60]. As their structure and properties differ significantly we have decided to present them in separate sections. The main groups of chemical compounds found in hops, their (skeleton) chemical structure, and typical representatives are collected in Figure 3. Detailed structures of the mentioned representatives are provided in the supplementary Figure S1.

Fractions consisting of soft resins (bitter substances) represent ~24% of dried hop cones and therefore significantly contribute to the taste of hop cones and consequently beer [8]. α-acids have been quite thoroughly studied as they form the larger portion of bitter substances. However, some studies have shown that β-acids exert even greater antimicrobial activity compared to α-acids [61]. 

Similarly to other plants, hop cones also contain a very diverse group of compounds called polyphenols. Beside polyphenols present in the majority of plants, hop cones also contain prenylflavonoids and multifidol glucosides that are almost exclusively found in hops, at least in significant quantities. It has been shown that members of the prenylflavonoid class of polyphenols exhibit several beneficial biological activities and could be therefore exploited as novel drugs for treating and preventing several diseases along with cancer [52,58,62]. Other polyphenols present in hops have also already been investigated as they are found in the majority of plants. Consequently, their biological features are known and certain polyphenols are already in the last stages of clinical trials as anticancer agents [24,63,64].

Last but not least, properties of hop essential oil components are also worth mentioning as potential natural healing substances. Unlike in the case of polyphenols, main constituents of hop essential oils are well-known substances present in a majority of plant essential oils. This, however, should only emphasize the importance of their existence in the plant kingdom. Meaning, that these compounds unquestionably play a crucial role in plants’ protection mechanisms. 

Equally important to treating and slowing down the development of prevailing diseases is their prevention. The food industry has already begun employing natural (plant) antioxidants as functional additives [65]. Authors [66] have confirmed the strong protective/antioxidative activity of hop cones for stabilizing lamb patties. Therefore, hops definitely exert the potential for exploitation in the food industry as a natural preservative. 

### 3.1. Soft Resins (Bitter Acids)

Due to the high content of biologically active compounds, the biological effects of hops foremostly refer to the mature female hop cones (flowerings) and their extracts. Bitter acids and xanthohumol were also found in male inflorescences: their concentrations are similar to those found during early female flowerings [67]. The presence of bitter acids and chalcones was also confirmed in the leaves of fully grown hops even if their levels were generally lower than in the hop cones and were strictly related to hop varieties [67]. The hop leaves also contain volatile compounds, but in much lower amounts than the hop cones (< 0.05%) [4]. Substances found in hop plant are commonly called resins. According to their (in)solubility in hexane, the division to soft and hard resins is generally accepted; soft resins being hexane soluble. Soft resins found in yellow powder secreted by lupulin glands are mainly lupulic acids, chemically di- or tri-prenylated phloroglucinol derivatives, and homologs [4,60,68,69]. Owing to their bitter taste, the term ‘bitter acids’ is adopted in the literature. Among soft hop bitter acids a division into two categories is adopted. α-acids (alpha lupulic acids or humulones) and their homologs represent the first category and the larger portion of soft resins, while the minor part represents the β-acids (beta lupulic acids) with homologs named lupulones [70].

The most important representatives of α-acids (Figure 3) are humulone (35–70% of total α-acids), cohumulone (20–65% of total α-acids), and adhumulone (10–15% of total α-acids). As their chemical structures are very similar, the names and quantities of representative β-acids are analogous; lupulone (30–55% of total β-acids), colupulone, and adlupulone. Other minor representatives of bitter acids include posthumulone/postlupulone, prehumulone/prelupulone, and adprehumulone [4]. The quantities of both types of acids and their homologs, however, may vary greatly as they depend on the hop variety, climate and cultivation conditions [8,71].

The content of α-acids in different hop varieties also determines the content of xanthohumol, the main polyphenol in hops [8]. When exposed to high temperatures (100–130 °C) and pH (8–10) as in the case of hop boiling during the brewing process, alpha-acids isomerize to iso-α-acids that next to oxidized hop acids—humulinones—predominantly to provide the bitter taste of beer [34,72]. As a majority of hops is grown for the brewing industry, its price is proportional to the alpha-acid content [60]. 

Kurasawa and colleagues [73] established that dried hop extracts, when administered orally, increase the gastric juice volume without affecting its acidity. On the basis of those experiments, Zanoli and Zavatti [4] suggested that the bitterness (the taste) of hops represents a crucial factor in inducing gastric secretion via the cephalic phase. Later, Walker and coworkers [74] confirmed that the bitter acids comprising of α-, β-, and iso-α-acids are potential key components promoting gastric acid secretion and upregulation of the CHRM3 gene expression.

The bitter taste promoting digestion is not the sole property that can be attributed to bitter acids. Sedative effects of hops have been recognized for centuries; however, not long ago, Schiller and coworkers [75] established that the main component of hops providing sedative effects is indeed the α-acids. Nonetheless, they clearly state that contributions of other fractions like β-acids and essential oils are also significant. Consequentially, preparations for fighting insomnia or sleep disorders consisted of hop cones alone or in combination with other sedative herbs, such as Valerian, have been on the market for at least a decade [8]. 

As beer mainly consists of water, it would not be stable without additives. Hops, especially bitter acids, not only contribute to its taste, but also stabilize the foam and, even more importantly, exhibit strong antimicrobial activity. The strong antimicrobial activity of β-acids mainly stems from their hydrophobic nature facilitating the interaction with microbial cell membranes [69,76].

On the other hand, it is presumed that ionophore properties of iso-α-acids represent the main mechanism for their antimicrobial action [35,72]. Moreover, Schurr and coworkers [72] propose humulinic acids, the hydrolytic product of α-acids and iso-α-acids, as novel tasteless food preservatives, according to their microorganism inhibitory action.

In addition to antimicrobial and sedative effects, a majority of hop compounds exerts a great variety of other health-promoting activities such as antioxidative [66,77], anti-inflammatory [76] and various anticancer effects. 

Chen and Lin [78] proposed that a pivotal mechanism for the chemopreventive action of hop bitter acids represents the induction of apoptosis. Many subsequent studies confirmed that bitter acids trigger apoptosis, but the full mechanism of this action still needs to be uncovered. However, it is known that apoptosis is induced via both pathways: intrinsic mitochondrial and extrinsic [61,78,79]. Bitter acids affect the intrinsic pathway by altering the Bcl-2 family of proteins, and the extrinsic by enhancing the expression of p38 that activates p53 and the TRAIL (Fas and FasL) death-receptor pathway [78,80]. 

In addition to apoptosis-inducing effects, bitter acids are also capable of inhibiting chemically-induced tumor promotion in vivo [81] and angiogenesis; β-acids are even more active than α-acids in inhibiting tumor development [8,61,82], reducing proliferation [81], and even inhibiting the growth of tumorigenic cells [68].

Finally, several in vivo studies [61,83,84,85] demonstrated that humulone, derivatives of α-acids such as iso-α-acids or rho-iso-α-acids and even hexahydro-β-acids inhibit various animal edema, thereby reducing inflammation. This activity probably stems from interactions with other important enzymes like COX, IKK (NF-kB), and Jun N-terminal kinase with mitogen-activated protein kinases (MAPKs), as was explicitly demonstrated in the case of humulone [84].

### 3.2. Polyphenols

One of the crucial roles in plant organisms: the protection from all kinds of external threads, has been ascribed to a diverse group of substances called polyphenols. Many of hop polyphenols are, therefore, abundantly found in other plants as well, however, the prenylflavonoid class is, to an extent (quantity), only present in the hop plant. Additionally, in hops, one can also find a group of metabolic products of already described bitter acids called multifidol glucosides. 

In this article we focus on the compounds encountered solely in hops, more substantial and detailed information on other polyphenols can be found in our review paper [24].

Lupulin glands secrete a mixture of prenylated, geranylated, oxidized, and/or cyclized chalcones along with bitter acids and volatile oils [69].

As polyphenols represent secondary metabolites they can be found in all plant parts. However, it was shown that the ethanol extract of hop cones has a 10-times greater content of phenolic substances than the hop leaves [33]. Presumingly, this could be the reason why hop leaves exert a lower antioxidant activity and were devoid of significant antimicrobial activity. Moreover, the authors also described that the phenolic profiles of the two parts of the hop plant were significantly different. It was suggested that a detailed identification of phenolic profiles is essential before exploitation of hop leaves as antioxidants [33]. 

Similar to the soft resins, the phenolic fraction of hop cones have been extensively studied. Xanthohumol, that represents more than 1% of dried hop cones is especially interesting and remains a topic of research for several applications [8]. 

For better transparency, polyphenols are presented separately in corresponding subclasses.

#### 3.2.1. Prenylflavonoids

Prenylflavonoids represent a class of flavonoids with at least one prenyl or geranyl substituent in the ring [49]. It was established that the prenyl substituent significantly alters the biological activity of corresponding flavonoids, likely due to the increased lipophilicity that improves the binding affinity towards biological membranes [49]. Except for the case of estrogenicity of genistein [78], the shared observation is that prenylation elevates the biological effect of nonprenylated moieties, both phloroglucinol derivatives and flavonoids [86,87,88].

It is presumed that the desmethylxanthohumol represents a precursor for the majority of flavonoids in hops [69]. Together with bitter acids, the content of desmethylxanthohumol and xanthohumol (XN) rises during the cone formation, which emphasizes their importance for the hop plant [67]. In addition, several other very important prenylflavonoids are found in hops, such as the most potent phytoestrogens 8-prenylnaringenin and 6-prenylnaringenin or the isomere of xanthohumol—isoxanthohumol.

Several in vitro [89,90,91] and in vivo rodent studies [92,93] undoubtedly reveal that even though prenylation increases the uptake of parent flavonoids into the digestive tract, enhanced in tissue accumulation results in diminished (lower) bioavailability of its prenylated counterpart. Rodent studies [92,93] demonstrated that naringenin accumulated in muscle tissue and quercetin in liver tissue. This, however, in the case of long-term dietary use (as supplements), requires attention to avoid deleterious effects of bioaccumulation in nontarget tissues as detoxification of prenylflavonoids from the blood seems much slower as their nonprenylated counterparts [88]. Clinical study of oral bioavailability of prenylflavonoids from hops revealed that even though 6-PN is significantly less bioavailable than 8-PN, it is equally effective in enhancing peripheral blood mononuclear cells viability [94].

Numerous studies have uncovered a great potential of xanthohumol as a new anticancer agent. Its capabilities extend from the prevention of cancer through all cancer stages. Additionally, xanthohumol possesses significant antimicrobial properties: of the five compounds isolated from hops, it was found the most effective against pathogenic fungi and it is even effective against malaria and HIV-1 viruses [8]. Moreover, we must not overlook that similarily to bitter acids xanthohumol exerts neuropharmacologically activity resulting in a sedative effect, reviewed in Karabin et al. [82]. 

Gerhäuser and coworkers [95] presumed that the chemopreventive properties of xanthohumol stem from its ability to inhibit metabolic activation of certain procarcinogens by cytochromes P450, like benzo[a]pyrene (BaP) and 2-amino-3-methyl-3H-imidazo[4,5-ƒ]quinoline (IQ). However, Plazar [49] showed that in rat liver slices (in vivo) xanthohumol does not inhibit the metabolic activation, but only protects against the genotoxicity of IQ and BaP, partially even against reactive oxygen species (ROS) and corresponding oxidative DNA damage. Several studies [95,96,97] also suggested that the induction of detoxification enzymes represents yet another chemoprotective mechanism of xanthohumol. This presumption was also rejected by Plazar [49], proposing that the protective mechanism could be ascribed to the inhibition of the cellular uptake of genotoxic IQ and BaP or that rather the metabolic products of XN (and not XN itself) are responsible for protection. An evidence supporting the first argument is that xanthohumol decreased the human recombinant DNA polymerase α activity in the MDA MB 435 human breast cancer cell line, thereby slowing down the (mutated) DNA replication [98].

In addition to the chemopreventive, sedative and antimicrobial activity xanthohumol also exhibits anti-inflammatory properties. At least a portion of this action originates in its ability to influence the IKK activity whether through the inhibition of TNF-induced IKK activation or through the suppression of the nuclear translocation of NF-κB by direct interaction with cysteine residues of IKK [99].

Several in vitro studies showed that XN can induce apoptosis by downregulating Bcl-2 or by acitvation of the caspase cascade and can even inhibit the growth of several cancer types like ovarian, breast, colon, prostate, hepatic, pulmonary cancer, and leukemia [82,100]. The second proposed mechanism of apoptosis triggered by xanthohumol is the induction of ROS [101]. The background of this effect was only recently revealed by Zhang and colleagues [102], who discovered that xanthohumol inhibits mitochondrial oxidative phosphorylation consequentially causing ROS generation and therefore inducing apoptosis of cancer cells. Additionally, the most prominent prenylflavonoid is involved in the inhibition of angiogenesis and metastasis as it was shown to inhibit microcapillary tube formation of HMEC-1 cells [8,82,103]. Xanthohumol was proven not only to be efficient against cancer but also against osteoarthritis [104], diabetes [105], and endometriosis [106].

All in all, various studies reported about xanthohumol’s antioxidative action, antimicrobial, sedative, anti-inflammatory, chemopreventive, and several other effects. A few mechanisms behind these effects have already been proposed; however, quite a few still need to be uncovered through in vivo studies and clinical trials.

Even though the isomer of xanthohumol—isoxanthohumol—was found to be less potent than xanthohumol in the majority of anticarcinogenic features, it was shown to be more anti-mutagenic and antiangiogenic, and even exhibits limited estrogenic activity [8,107]. The metabolism of xanthohumol does not end in isoxanthohumol the activation by intestinal microflora or cytochrome P450 enzymes yields one of the most potent phytoestrogens—8-prenylnaringenin (8-PN)—capable of binding to α- and β-estrogen receptors [4,8,87]. Rad [108] showed that even in high doses (750 mg) 8-PN is well tolerated, the absorption is fast, and the stability is good. Clinical tests confirm that 8-PN represents a promising novel therapeutic agent for the treatment of menopausal and post-menopausal symptoms [58]. Even though standardized hops extract containing 0.42% of 8-PN did not stimulate the growth of methyl-nitrosourea induced mammary cancer and proliferative events in the normal mammary gland of Wistar rats, some safety issues remain regarding the potential adverse effects associated with a long-term consumption of phytoestrogens [58,109]. However, opposed to the hormone-replaced therapy intake of hop and beer polyphenols, the above-mentioned method represents a safer and more effective treatment for menopausal and postmenopausal women [77,110]. It has been shown that regular polyphenol consumption reduces vasomotor symptoms and osteoporosis and relieves other common symptoms of menopause [77].

Similarly to isoxanthohumol, 8-prenylnaringenin exhibits several anticarcinogenic activities like strong antiangiogenic effect and the inhibition of survival and proliferation of estrogen-responsive cells by interfering with the PI3K pathway [111]. 8-prenylnaringenin was shown to be a better inhibitor of the metabolic activation of IQ [82], therefore confirming Plazar’s hypothesis [49] that metabolic products of xanthohumol contribute more to its chemoprevention than the xanthohumol itself. Both XN and 8-PN also proved to be strong inhibitors of NF-κB activation in microglial cell lines, thereby possessing the ability to modulate immune responses in the nervous system [82].

Delmulle and coworkers [112] discovered that hop prenylflavonoids (xanthohumol, isoxanthohumol, 8-prenylnaringenin, and 6-prenylnaringenin) induce the nonapoptotic, caspase-independent form of cell death. For hop-derived prenylflavanones the form of cell death seems to be autophagy, due to the increased formation of vacuoles, however, for XN this was not observed. All in all, all hop-derived prenylflavonoids, including desmethylxanthohumol, proved to trigger some sort of cell death [8]. Like xanthohumol, desmethylxanthohumol also inhibited the growth of leukemia cells [113] and 6-prenylnaringenin showed a significant antifungal and antibacterial effect [112,114].

#### 3.2.2. Catechins

According to its content in hop cones, the class immediately following the prenylflavonoids is flavanols, also called catechins, as well as their polymers proanthocyanidins and condensed tannins [8]. The most abundant in hop cones is flavanol (+)-catechin— the third most abundant of the individual compounds [8]. This flavanol is predominantly found in hawthorn berries and leaves and became one of the active substances in herbal preparations for strengthening the cardiovascular function possessing both antioxidative and vasodilative features [8].

Other abundant flavanols are (−)-epicatechin and (+)-gallocatechin [82]. Listed catechins are also important tea components, especially of green tea, therefore studies mainly report the properties of tea catechins. 

In human prostate and breast cancer cells, flavanols catechin and epicatechin have exhibited antioxidative and antiinflammmatory effects as well as the inhibition of telomerase [115]. Along with various other polyphenols, they time- and dose-specifically decrease the proliferation of breast and prostate cancer cells; are able to interact with estrogen and androgen receptors, even at nanomolar concentrations; and lower the amount of NO species by decreasing its secretion and inhibiting its production [116]. Epicatechin, along with other tea catechins, additionally suppresses the growth of various types of cancer cells and by inhibiting apoptosis in the PC12 cells also exerts neuroprotective effects, moreover, it acts as an anti-aging agent [116,117]. On the other hand, catechin significantly (70%) inhibits intestinal tumor formation and suppresses FAK consequentially decreasing mobility and lowering metastasis [116].

In nature, we almost exclusively find mixtures of substances, not individual compounds and interactions emerging from mixtures can enhance the effects of individual components or even give rise to new effects. In the case of hop plants, it was shown that a mixture of hop proanthocyanidins is indeed more antioxidative than individual flavanols and proanthocyanidins [8]. Logically, a mixture of a variety of hop flavanols can also provide a protection from a broader spectrum of microorganisms than only a single compound is capable of.

#### 3.2.3. Flavonols

Flavonols are yet another class of flavonoids. The most prominent members of flavonols are quercetin and kaempferol. Both compounds can be found in various fruits and vegetables not only in hops. In the literature, quercetin and kaempferol are reported as one of the most potent antioxidants [8,82].

Plants, including hops, mostly contain flavonols as glycosides [18]. Their bioavailability depends on the glycosidic part. In the case of quercetin, glucosides represent the most bioavailable form (even more than aglycone). Unfortunately, the least bioavailable form of quercetin—rutin—represents the most common form of quercetin in hops [8,18]. Fortunately, several approaches exist for enhancing the bioavailability of polyphenols, the most promising ones can be found later in the concluding section.

In vitro and even in vivo studies have established that quercetin downregulates cell survival and proliferative proteins as well as induces apoptosis and dose-dependently impacts cell growth [118,119]. The apoptosis may be induced partially through hyperacetylation of histones H3 and H4 reported for human leukemia cells and lung cancer cell lines as well as partially by modulating the expression and activity of Mcl-1: antiapoptotic proteins belonging to the Bcl-2 family [120,121]. Quercetin also belongs among a few polyphenols that can induce autophagy by downregulating HSP72 [120]. Moreover, quercetin possesses the ability to cross the cellular/nuclear membrane and is consequentially capable of engaging in the epigenetic regulation probably owing to its lipophilicity [120]. Suppression of COX-2 by blocking multiple transactivators and p300 signaling is finally enabling quercetin to partake in the anti-inflammatory processes [120]. Like epicatechin, it is perceived as an anti-aging agent [117]. 

Quercetin is not only able to act on its own but it additionally potentiates the effect of established chemotherapeutic drugs as was for example shown in the case of fludarabine for treating chronic lymphocytic leukemia [121].

Quercetin and kaempferol are also considered good chemopreventive agents for their ability to activate (detoxification) phase II enzymes [82]. However, these presumptions still need a confirmation through in vivo studies. 

Among several polyphenols, quercetin and kaempferol dose-dependently inhibit the growth of various cancer cells showing different sensitivity between the cell lines [122]. Additionally, in water and methanol extracts of hops significantly inhibited protein kinase C and, therefore, represent possible silencers of oncogene expression. Moreover, hop water extracts, containing quercetin and kaempferol glucosides, have also shown the ability to inhibit histamine release and thereby type-I allergic reactions [123]. 

Analogously to quercetin, kaempferol also proved capable of inducing hyperacetylation of histone H3 complex in human liver and colon cancer cell lines, therefore reducing cell viability and proliferation rate [120]. Finally, in silico molecular docking and in vitro profiling have revealed kaempferol as a pan-inhibitor of human HDACs of classes I, II, and IV, meaning that it can also impact cell differentiation and apoptosis [120].

#### 3.2.4. Multifidol and Multifidol Glucosides

The name multifidol was given by Kosasi and coworkers, as they found compound (2-methyl butyryl)phloroglucinol in the latex of the shrub *Jatropha Multifida* that is used in folk medicine for the treatment of infected wounds, skin infections, and scabies [124]. In ethanolic hop extracts Bohr and colleagues [125] subsequently identified four acylphloroglucinol-glucopyranosides. The first 1-(2-methyl propanoyl)phloroglucinol-glucopyranoside had been found in hops before. Other were multifidol glucosides, 1-(3-methyl butyryl) phloroglucinol, and 5-(2 Methylpropanoyl) phloroglucinol. Consistently with the nomenclature of hop bitter acids, the substances were named co-, ad-, n-, and co-iso-multifidol glucosides. Discovered phloroglucinols were tested for inhibitory COX-1 activity and all listed substances exhibited corresponding anti-inflammatory activity [125]. Their potential, however, decreases with increasing length of the acyl side chain [125]. The authors also noted that the acyl side chains of identified phloroglucinols are identical to hop iso-α-acids, therefore, representing intermediates in the biosynthesis of hop bitter acids [125].

Akazawa and colleagues [86] later isolated a new phloroglucinol derivative 5-deprenyllupulonol C that among certain other phloroglucinols, exhibits inhibitory effects on Epstein–Barr virus early antigen (EBV-EA) induction and 12-O-tetradecanoyl phorbol-13-acetate (TPA)-induced inflammation in mice. Phloroglucinol derivatives from hops likely carry a great anticarcinogenic potential as phloroglucinol derivative 2,4-bis(4-fluorophenyl acetyl)phloroglucinol induces concentration-dependent cell death and antiproliferation in three types of glioma cells but not in primary human astrocytes [126].

Dresel and coworkers [127] determined human recognition threshold concentrations and the lowest of a total 11 bitter tastants from the hop hard resin fraction was awarded to co-multifidol glucopyranoside at a concentration as low as 5 μmol/L. All in all, even minor hop compounds may be significant carriers of taste, not only from the soft resin, but also from the hard resin hop fraction [127].

#### 3.2.5. Phenolic Acids: Ferulic Acid

Hop cones, also contain a certain amount of ferulic acid belonging to the hydroxycinnamic acids from the class of phenolic acids. For quite a while, ferulic acid was unjustifiably neglected as it possesses several health-promoting activities. It is similar to other highly antioxidative polyphenols as it prevents lipid peroxidation, apoptotic cell death of healthy cells, and is an effective and multifunctional free radical scavenger [82,128]. It slows down the degradation of iso-α-acids and therefore partially prevents the spoiling of beer [34]. Unlike many polyphenols, the ferulic acid in its free form is very efficiently absorbed as approximately 50% of the ingested dose gets recovered in urine [18,128]. However, in cereals, the esterified ferulic acid is only poorly absorbed [18]. Ferulic acid is able to ameliorate toxicity of certain chemicals, carcinogenic agents, ionizing radiation, and is even a strong UV absorber [125,128]. Interactions with several enzymes enable the ferulic acid to act as an anti-inflammatory, antiapoptotic, and anticarcinogenic agent. In cancer cells, it is shown to decrease cell viability and colony formation while it inhibits cell migration and invasion [128]. Zhang and coworkers [129] therefore proposed ferulic acid for breast cancer therapy. Owing to its antidiabetic, hepatoprotective, cardioprotective, and neuroprotective features, ferulic acid should also be used for treating other diseases like diabetes, Alzheimer’s, cardiomyopathic disorders, and many others [128]. Compared to flavonoids that are a strong antimicrobial agent, ferulic acid exerts weaker activity; however, it still has significant activity against several Gram positive bacteria such as *S. aureus*, *L. monocytogenes* and Gram negative bacteria, including *Pseudomonas aeruginosa* [82]. Finally, we must not overlook important biological effects of ferulic acid dimer–curcumin–widely used as a food preservative and yellow coloring agent in foods, drugs, and cosmetics [130].

#### 3.2.6. Stilbenes: Resveratrol

From the revelation of (the French paradox), resveratrol has become one of the most known polyphenols. Copious studies, even in vivo, have reported about its anti-inflammatory and several anticancer effects. In various cancer types, resveratrol inhibits of tumor formation and growth, angiogenesis, metastasis, and induces apoptosis. One of the possible mechanisms of resveratrol’s anticancer activity, at least in breast cancer cells, is its ability to induce the expression of SERCA3 gene fundamental for maintaining the intracellular (Ca^2+^) homeostasis, which results in a decreased breast cancer cell viability and triggered apoptosis [131]. Zeng and coworkers [132] also suggested that resveratrol inactivates PI3K/Akt signaling by upregulating bone morphogenetic protein 7 (BMP7) gene. In addition, resveratrol possesses several features for the prevention or improvement of cardiovascular diseases [24,133]. Moreover, it is perceived as an anti-aging agent [117]. Consequently, resveratrol has become a part of numerous patents for therapeutic, cosmetic, and nutraceutical applications [133]. All in all, several dietary supplements of resveratrol can be found on the market in various formulations to enhance its poor absorption and bioavailability [133].

### 3.3. Essential Oils

Essential oils, as is already evident from the name, represent the essence of the plant, meaning its distinctive aroma. Hop aroma has always intrigued the mankind and it represents the significant portion of beer aroma. It is actually the quest for a better beer aroma that evidently brought so many hop varieties [4,57]. Even though this subject has been in the center of scientific and brewer’s attention for centuries, the whole list of substances contributing to the hop aroma and consequentially to beer taste is still not completed. Following the bitter fraction, hop essential oil compounds are also secreted from lupulin glands of female plants [82]. According to the basic molecular structure hop, essential oils consist of three fractions. In the first hydrocarbon fraction, one can find monoterpenes, sesquiterpenes, and aliphatic hydrocarbons. The second fraction contains oxygenated compounds like terpene and sesquiterpene alcohols. In spite of the fact that many believe that the last fraction of sulfur-containing compounds does not contain important biologically-active molecules, this may not be entirely true. Compounds from the sulfur fraction moreover proved to be important contributors to the hop and beer aroma and prevent its spoiling [34,134]. 

Clustering analysis of essential oils from 25 different hop varieties has shown that the highest amounts, accounting for 47.1 to 89.3% of the oils, represent β-myrcene, α-humulene, β-caryophyllene, caryophyllene oxide, and humulene epoxide II [135]. This is almost completely consistent with the study of Ligor and coworkers [22] study who listed the most important components of hop aroma as myrcene, α-humulene, β-caryophyllene, and β-farnesene. A newer study where comparative aroma extract dilution analysis was performed on the special flavor hop varieties Huell Melon and Polaris determined myrcene, (3R)-linalool, and 2- and 3-methyl butanoic acid as important variety-independent hop odorants and found (1R,4S)-Calamenene—a new odor-active compound in hops [57]. Considering the division into three fractions, the majority of the hydrocarbon fraction is made up of monoterpenes α- and β-pinene, myrcene, and limonene as well as sesquiterpenes α humulene, β-farnesene (not in all hop varieties), β-caryophyllene, α- and β-selinene, and γ-muurolene [8,82]. During the ripening, processing, and storage oxidation processes occur and yield the oxygenated fraction containing linalool, geraniol, caryophyllene oxide, 2-methyl-3-butene-2-ol, and farnesol forms [8,82].

However, the content of a specific substance in essential oils not only depends on the cultivation conditions, storage, and processing, but the extraction and analytical process importantly impact it as well [22,71]. 

An important (hop) essential oil component monoterpene myrcene has two sides. According to the International Agency for Research on Cancer (IARC), myrcene, contained in certain popular beverages, is regarded as a potential human carcinogen; however, it is clearly stated that this fact still needs to be further evaluated [136]. On the other hand, both myrcene and linalool significantly inhibited the genotoxicity of 2-amino-1-methyl-6-phenylimidazo[4-5-b]pyridine and only myrcene, even though less efficiently, inhibited the toxicity of 2-amino-3-methylimidazo[4,5-f]-quinoline [137]. β-myrcene has also shown to be a potent dose-dependent TNF-α inhibitor, stronger than α-pinene and d-limonene, through the phosphorylation of the inhibitor of κB kinase and matrix metalloproteinase-9 (MMP-9) gene expression [138]. Moreover, β-myrcene also inhibited invasion of MDA-MB-231 cells (breast cancer cells) induced by TNF-α [138]. More studies argue in favor of positive health effects of myrcene than of its carcinogenicity, therefore, we cannot exclude it from the list of potential anticancer agents.

Myrcene, extracted together with other essential oil compounds from used hops, exhibited the highest repellency towards insect *Rhyzopertha dominica* (RD50 = 0.27 A mu M cm^−2^), whereas for yet another insect degrading stored foods *Sitophilus granarius*, limonene was the most effective repellent [139]. Therefore, not only fresh hops but even left-over hops from breweries can be effective at least as an insect repellent, protecting stored foods [139].

The major contributor to the hop aroma from the essential oil fraction is presumably the monoterpene β-pinene found also in rosemary, parsley, dill, rose and other essential oils [107]. It was shown that both α-pinene and β-pinene generated a substantial synergistic effect with the Paclitaxel drug for treating non-small cell lung carcinoma [140].

Among sesquiterpenes found in hop essential oils (and other essential oils from clove, piper, and hemp), β caryophyllene seems to be the most important regarding biological effects. Its spiciness, partially coming from the antagonistic action towards the cannabinoid receptor (CB), is actually more representative of piper and clove aroma than hop [107]. Several in vitro studies have shown that β caryophyllene and its oxide possess significant anticancer activities, affecting growth and proliferation of various cancer cells [141]. Especially β-caryophyllene oxide proved to alter several key pathways of cancer development, such as MAPK, PI3K/AKT/mTOR/S6K1 and STAT3 pathways [141]. As β-caryophyllene activates only CB2 and not CB1, it carries a potential as a novel natural analgesic drug. Additionally, both compounds enhance the efficacy of standard drugs by augmenting their concentrations inside tumorigenic cells [141]. All in all, these compounds represent the future of cancer treatment with natural substances. In the oxygenated fraction, one can also find 2-methyl-3-butene-2-ol, whose concentration increases during the storage of hops [8]. This compound is believed to be the most responsible for the calming effect of hop essential oils [8].

Humulene (α-caryophyllene) found in mixtures together with β-caryophyllene represents an important sesquiterpene substance providing the distinctive hoppy aroma to the beer and exhibiting some mild corticosteroid effects. Humulene gets epoxidized during the brewing process, however, it is the hydrolyzed form that provides its taste, not the epoxidized [107]. 

The minor fraction, representing less than 1% of hop essential oils, are sulfur-containing compounds like thiols, sulfides, polysulphides, thioesters, thiophenes and terpene derivatives, which may also contribute to the beneficial biological effects exerted by hops [142].

Thioesters have been also detected in thermally not processed unkilned hops, suggesting that they are naturally occurring components of hop essential oils, although present in lower concentrations than after heat processing that causes their additional formation [142]. A study of Guadagni and coworkers [143], on the odor contributions of 25 main chemical components of hop essential oils from Bullion hops, has allocated S-methyl hexanethioate on the second place regarding its contribution (4–8%) to the hop aroma. Moreover, S-methylthiomethyl thioesters have been uniquely found in hops [142].

More than forty-one different volatile polyfunctional thiols, detected in various hop cultivars, also represent key contributors to the hop aroma in beer as their content rises during drying and brewing processes [142,144]. Polyfunctional thiols have been appreciated and widely studied in the enology field as the compounds providing desirable odor notes like rhubarb, citrus, passion fruit, or blackcurrant bud carried by 3-mercaptohexan-1-ol (3MH), 3-mercapto hexyl acetate, 4-methyl-4-mercaptopentan-2-one (4MMP), 3-sulfanyl-4-methyl pentane-1-ol, 3-sulfanylhexan-1-ol, and others [134,144]. In addition to releasing pleasant aromas, 3-mercaptohexan-1-ol also inhibits epigallocatechin gallate (EGCG) oxidation, thereby providing the necessary stability for EGCG to retain its beneficial biological activity [145].

Roland and coworkers [134] investigated in which form one can find thiols, especially 3MH and 4MMP in hops. For 4MMP the free-form represents the most important fraction (up to 95–100%) with precursors found in trace amounts only or even completely absent. However, surprisingly, the contrary was found for 3-mercaptohexan-1-ol, where more than 99% were allocated to its precursors: S-cysteinylated and S-glutathionylated conjugates. In a majority of hop varieties, glutathionated 3MH represents more than 80% of the total amount, only in Barbe Rouge hops the proportion is 50:50. Free 3MH represents less than 1% in all hop varieties, although some studies reported up to 7.1% of the free fraction [134]. In plants in general and also in hops, it is more common to encounter cysteine-S-conjugates as they represent the final product of the glutathione detoxification pathway [144]. Since from glutathione-S-conjugates free glutathione can be released, consumed thiol precursors from hops may importantly contribute to the organism’s detoxification processes and therefore prevent the oxidative stress and other diseases like cancer. 

To conclude, organosulfur compounds found in hop essential oils can be beneficial through at least three different ways. Some are carriers of the aroma and flavor and can thereby importantly affect our mental and physical state according to the foundations of aromatherapy. Others prevent oxidation/degradation of important biologically active molecules like EGCG. Some may again release glutathione and can, therefore, contribute to the detoxification of organism, especially from numerous tumorigenic substances surrounding us. Last but not least, even compounds not biologically active on their own can still bring about synergistic effects and therefore enhance health-promoting activities of other more active substances contained in hops or even provide new effects that none of these compounds feature on their own. 

An overview of hop compounds, applied extraction methods, and described biological effects is provided in Table 1. 

## 4. Conclusions and Future Perspectives

Extracts obtained from brewing wastes may also serve as important dietary sources of polyphenolic compounds. Almost all the parts of the hop plant contain many nutritional and pharmaceutical properties. Nevertheless, according to the so-called “biorefinery” concept, the production of value-added bioproducts like phytochemicals from food industry wastes should not only help to lower the negative environmental impact, but should also add economic value to the brewing products hence progressing towards the sustainable development of the beer industry. The biorefinery concept has been already integrated into modern day brewing technological advances which tend to reduce the amount of waste produced and to generate useful materials from the by-products of brewing.

Some waste streams, attained during the beer brewing process, contain high amounts of sugars, lignin, and essential amino acids [158]. Moreover, waste brewery biomass which includes spent grain, spent yeast, and spent hops/hot trub, is rich in bioactive phenolic compounds with antioxidant activity, which can be recovered by various extraction methods, including solid–liquid extraction, microwave-assisted extraction, enzymatic reactions, and alkaline reactions. 

In order to optimize the recovery of polyphenols, the research on novel extraction techniques (maceration, ultrasound, and microwave-assisted extraction) and separation techniques in combination with alternative solvents is gaining increasing interest. The polyphenolic fractions are tested for their chemical and biological properties. On the other hand, the biomass remaining after the extraction or processing of biological materials, such as brewers’ spent grain, still contains a number of valuable components and therefore represents a source of alternative fuels as well as the source of chemical compounds. This certainly represents an additional challenge to obtain value added products from the low-value streams. An additional advantage is derived from the generally displeasing fact that polyphenols may unfavorably influence beer stability. According to the biorefinery concept, crude extracts of phenolic compounds with high antioxidative activity can be obtained from the brewery waste stream produced during regeneration of polyvinylpolypyrrolidone (PVPP) resins used to clarify beer. The concentration of haze-active proteins and polyphenols is reduced by the adsorption with PVPP [159]. Huge amounts of these compounds are therefore present in the waste stream, which contains large amounts of polyphenols, and therefore represents a promising alternative and an economical source of natural antioxidants and antimicrobials with various applications in the food industry. Food industry is aiming to intensify also the studies regarding organoleptic properties of beer related to packing and storage. Recent research shall be shifted towards investigation of absorption kinetics of hop volatiles into different crown cork liner polymers and can coatings due to the issue of flavor scalping, connected to the loss of aroma substances in food containers. This phenomenon is the result of their migration into the packaging materials. Information related to the absorption of aroma-active beer constituents by different “modern” PE beer bottle closures and can coatings, as well as the concurrent uptake rate of hop volatiles into crown cork liners, is thus far lacking in the published literature. Substances absorbed by the polymers are usually determined by the supercritical fluid extraction/gas chromatography method. Polypropylene absorbs most aromas: higher amounts are absorbed by low-density polymers. 

In the case of low-density polyethylene (LDPE) liners with oxygen-scavenging functionality, oxygen-barrier liners made up of high-density polyethylene (HDPE) or linear polymers from a different manufacturer had no significant effect on the composition of hop volatiles in beer after prolonged storage. It has also been demonstrated that can coatings are able to absorb hop volatiles in a similar pattern as crown corks although to a lesser extent. Consequently, significantly higher percentages of myrcene were found in the beer [160].

Certain hop extracts are already in general use, especially for providing sedative and estrogenic effects. Many more could potentially be used as several in vitro studies point out that a number of compounds isolated from hops exert significant biological activities. However, in vivo studies at physiologically attainable concentrations to confirm these activities are still relatively rare. It is not the main question whether these compounds really possess features enabling them to impact the enzymatic action and/or cellular processes, but rather if these active substances reach the target cells in sufficient concentration and form to provoke/reach the desired effects. We must also consider that the action of many substances differs greatly when applied in different matrixes. After the ingestion of a given substance, its metabolism plays a crucial role and importantly impacts its absorption. The majority of polyphenols including prenylflavonoids, with the notable exception of catechins, is poorly absorbed [24,89,90,91,92,93] and therefore have low bioavailability. However, there are several approaches to improve the absorption or to reach the desired effect at lower doses. Next to nanoencapsulation [119,161,162], oligoglycosylation in the case of quercetin [163] and microrization of hesperetin [164] exhibited a great potential, even in an in vivo human study, for increasing the bioavailability. Producing the mixtures/combinations of biologically active substances represents another practiced approach with several important advantages. The knowledge on encapsulation of extracts, including plant extracts such as hops, is still relatively scarce. Therefore, there is an idea either to develop suitable techniques or to modify the existing formulation techniques [165] which would yield extract formulations with a high bioavailability. To examine their biological effects, further in vitro and in vivo studies would be required. The latter can prevent the potential high dose toxicity or improve the tolerance of already established drugs and therapies and additionally bring about potential synergistic effects. Synergistic effects due to the lower needed concentrations not only prevent the negative side effects of compounds and the potentially developed resistance, enhance their beneficial effects but also bring new effects that none of the compounds, like the truly neglected sulfur-containing hop compounds, may initially possess on their own. For a great number of substances, their biological activity is yet to be determined. On the other hand, because hops have been traditionally used in brewing the most important constituents of the ’hoppy’ aroma have been profoundly investigated. As the scent represents one of our strongest senses and the only one directly connected to the brain, even compounds representing a really minor portion of the hops can evoke certain positive psychological and physiological effects. In plants one can only find mixtures of compounds, which are capable of numerous strong health-promoting activities, the majority have not yet been found/studied, this can help to prevent the formation and even start the healing process of several common diseases like cancer. 

In the search for novel drugs before turning to potentially hazardous laboratory experiments, one should exploit the swiftly developing computer technology. Computers not only improve laboratory work plans but also predict several properties of single molecules or entire molecular systems under various conditions and even carry our simulations of chemical reactions and of more and more biological cellular processes. Cancer really represents plague of the modern era and investigating cancerogenic substances without physically exposing oneself to them certainly poses a significant advantage. For that, it is reasonable to investigate the antigenotoxic potential of natural compounds found in hops also through quantum chemistry-based reaction simulations. Such simulations enable uncovering the mechanisms of action of known antigenotoxic agents and even reveal new potential antigenotoxic agents. In silico quantum–chemical methods used by our laboratory carry a great potential as they are safe, effective, relatively fast, cost-effective and more importantly exhibit a good correlation with the laboratory experiments [166,167,168]. In order to explore the antigenotoxic potential of a specific substance, we first obtain geometrically optimized structures of the species of interest like carcinogenic substance, DNA bases, or potential antigenotoxic agent. Next, we locate, with several quantum–chemical methods and basis sets, reactant, and transition state structures for the reaction of our interest in order to determine the energetic barrier—activation energy. In order to find potential antigenotoxic agents, it is necessary to perform such calculations for the reaction of a carcinogenic substance with DNA and with potential antigenotoxic substances so as to get comparison which of the reactions is energetically more favorable and therefore plausible. The reaction of scavenging (antigenotoxic) compound with the carcinogenic substance must be faster/energetically more favorable than the reaction between carcinogen and the DNA in order to prevent the genotoxicity caused by the carcinogen. 

**Figure 4:**
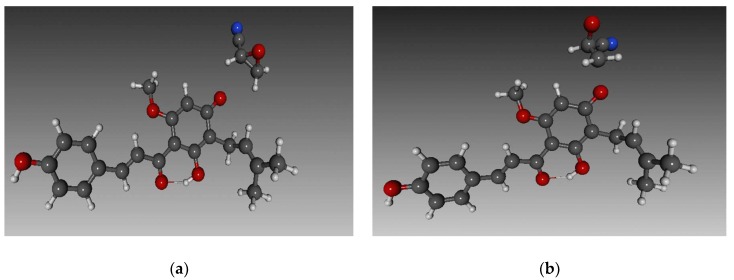
Quantum chemical model of (**a**) the reactant state and (**b**) the transition state of the reaction between xanthohumol and cyanoethylene oxide acquired according to the Hartree–Fock method and 6-31G(d) basis set [171]. Carbon atoms are depicted in gray, oxygen atoms in red, nitrogen atoms in blue, and hydrogen atoms in white.

For obtaining and optimizing structures the Gaussian Suite of Programs [169] was used. The analysis was performed with the Molden program [170], which also enables capturing frames to obtain pictures as seen in Figure 4 where the reactant and the transition state of the reaction between xanthohumol (polyphenol found in hops and known antigenotoxic agent) and cyanoethylene oxide—cytochrome P450 2E1 metabolized form of food carcinogen acrylonitrile [171], acquired using the Gaussian 09 suite of programs [172] according to the Hartree–Fock method and 6-31G(d) basis set presented. Xanthohumol was chosen as its antigenotoxic activity was already confirmed through in vivo and in vitro studies and simulation of its reaction with cyanoethylene oxide, a typical and common epoxy-type chemical carcinogen with confirmed carcinogenicity, can uncover xanthohumol’s antigenotoxic mechanism of action. Simulations of such reactions, like the one in Figure 4, between hop compounds and carcinogenic substances enable us to reveal the mechanism of known antigenotoxic agents and even find new potential antigenotoxic agents through in silico quantum–chemical methods. 

## Figures and Tables

**Table 1:** Hop compounds, applied extraction methods, and described biological effects.

Class of Compounds	Compound	Extraction Method	Health Effects	References
**α-acids**	humulone	supercritical CO_2_ extraction, *T* = 40–50 °C and *P* = 150–400 bar, accelerated solvent extraction	promotion of gastric acid secretion, sedative effects, antioxidative action, apoptosis-inducing, inhibition of tumor promotion, inhibition of angiogenesis, reduction of proliferation, reduction of inflammation, antimicrobial effects	[8,61,66,74,78,81,82,84,146,147]
**β-acids**	lupolone	supercritical CO_2_ extraction, *T* = 40–50 °C and *P* = 150–400 bar, accelerated solvent extraction	promoting gastric acid secretion, sedative effects, antimicrobial, anti-inflammatory, antioxidative effects, induction of apoptosis, inhibition of tumor promotion, inhibition of angiogenesis, reduction of proliferation, inhibition of tumor growth	[8,61,66,69,70,74,75,76,78,79,80,81,82,147]
**hexahydro-β-acids**		supercritical CO_2_ extraction, *T* = 40–50 °C and *P* = 150–400 bar, accelerated solvent extraction	reduction of inflammation	[61,83,84,85]
**iso-α-acids**		supercritical CO_2_ extraction, *T* = 40–50 °C and *P* = 150–400 bar, accelerated solvent extraction	promoting gastric acid secretion, antimicrobial effects, reducing inflammation	[61,72,74,83,84,85,147]
**rho-iso-α-acids**		supercritical CO_2_ extraction, *T* = 40–50 °C and *P* = 150–400 bar accelerated solvent extraction	reducing inflammation	[61,83,84,85,147]
**Polyphenols/prenylflavonoids**	xanthohumol	conventional extraction with polar solvents (ethanol, methanol), ultrasound extraction, microwave extraction, supercritical CO_2_ extraction, conditions of extraction, temperature 50 °C, and pressure 290 bar	anti-inflammatory, antimicrobial, sedative effects, protection against genotoxicity, slowing down (mutated) DNA replication, induction of apoptosis, inhibition of angiogenesis, inhibition of metastasis, anti-arteriosclerotic, antidiabetic, anti-endometriotic effects	[8,49,82,98,99,100,101,102,148]
	isoxanthohumol	supercritical CO_2_ extraction, conventional extraction with solvents of a high polarity (MeOH, EtOH), pressurized liquid (water) extractions, pressure 10.68 MPa and temperatures 50 °C, 100 °C, 150 °C and 200 °C; extraction time 30 min	Anti-mutagenic, antiangiogenic, estrogenic activity	[8,104,148,149]
	8-prenylnaringenin	supercritical CO_2_ extraction, conventional extraction with solvents of a high polarity (MeOH, EtOH)	strong estrogenic activity, chemopreventive effects, a strong inhibitor of NF-κB activation	[4,8,82,87]
**Polyphenols/** **prenylflavonoids**	desmethylxanthohumol	supercritical CO_2_ extraction, conventional extraction with solvents of a high polarity (MeOH, EtOH)	inhibition of leukemia cell growth	[8,135]
	6-prenylnaringenin	supercritical CO_2_ extraction, conventional extraction with solvents of a high polarity (MeOH, EtOH)	significant antifungal and antibacterial effects	[8,148,149]
**Polyphenols/** **catechins**	(+)-catechin	Conventional extraction, aqueous and pure organic solvents (acetone, ethanol, methanol, acetonitrile, water), different temperatures (60, 80, 95, and 100 °C), optimal solvents are acetone and acetonitrile	Antioxidative, vasodilative, anti-inflammatory effects, inhibition of telomerase, decreasing proliferation of breast and prostate cancer cells, interaction with estrogen and androgen receptors, inhibition of intestinal tumor formation, decreasing mobility, lowering metastasis	[8,115,148,150]
	epicatechin	Conventional extraction, aqueous and pure organic solvents (acetone, ethanol, methanol, acetonitrile, water), different temperatures (60, 80, 95, and 100 °C)	Antioxidative, antiinflammmatory effects, inhibition of telomerase, decreasing proliferation of breast and prostate cancer cells, interaction with estrogen and androgen receptors, suppressing the growth of various cancer cells	[115,116,150]
**Polyphenols/** **flavonols**	quercetin	Conventional extraction (ethanol and methanol) at moderate to elevated temperatures (50 to 80 °C), microwave assisted extraction and ultrasound assisted extraction	strong antioxidant activity, downregulation of cell survival and proliferative proteins, apoptosis-inducing, reducing cancer cell growth, induction of autophagy, ability to engage in epigenetic regulation, anti-inflammatory effects, a good chemopreventive agent, inhibition of cancer cells growth, inhibition of histamine release	[8,82,118,119,120,121,122,123,150]
	kaempferol	Conventional extraction with organic solvents of high polarity	strong antioxidant activity, a good chemopreventive agent, inhibition of cancer cells growth, inhibition of histamine release, reduction of cell viability and proliferation rate, impact on cell differentiation and apoptosis	[8,82,120,122,123,150]
**Multifidol and multifidol glucosides**		Conventional extraction with petroleum ether	anti-inflammatory effects, probable anticarcinogenic activity	[125,151]
**Polyphenols/** **phenolic acids**	ferulic acid	Non-pressurised alkaline hydrolysis (0.5 M NaOH) and pressurized solvents (0.5 M NaOH, water, ethanol, and ammonia)	highly antioxidative, amelioration of toxicity of several chemicals and carcinogens, anti-inflammatory, antiapoptotic effects, anticarcinogenic agent, decreasing cell viability and colony formation, inhibiting cell migration and invasion, antidiabetic, hepatoprotective, cardioprotective, neuroprotective, antimicrobial effects	[82,116,128,152]
**Polyphenols/** **stilbenes**	resveratrol	maceration at room temperature, extraction at elevated temperature, fluidized-bed extraction, Soxhlet extraction, microwave-assisted extraction, and accelerated solvent extraction, pressurized solvents (0.5 M NaOH, water, ethanol, and ammonia)	anti-inflammatory effects, inhibition of tumor formation and growth, antiangiogenic, antimetastatic activity, induction of apoptosis, inactivation of PI3K/Akt signaling, prevention or improvement of cardiovascular diseases	[131,132,133,153]
**Essential oils/monoterpenes**	myrcene	water steam distillation, supercritical fluid extraction with CO_2_	inhibition of genotoxicity, TNF-α inhibitor, an inhibitor of MDA-MB-231 cell invasion, good insect repellent	[137,138,139,154]
	linalool	water steam distillation, supercritical fluid extraction with CO_2_	inhibition of genotoxicity	[137,154]
	limonene	water steam distillation, supercritical fluid extraction with CO_2_	good insect repellent	[139,154]
	β-pinene	water steam distillation, supercritical fluid extraction with CO_2_	synergistic antitumor effects with the Paclitaxel drug	[140,154]
**Essential oils/sesquiterpenes**	β-caryophyllene	supercritical fluid extraction with CO_2_	affecting growth and proliferation, altering several key pathways of cancer development, analgesic effects, enhancing the efficacy of standard drugs	[141,154]
	β-caryophyllene oxide	supercritical fluid extraction with CO_3_	affecting growth and proliferation, altering several key pathways of cancer development, enhancing the efficacy of standard drugs	[141,154]
	2-methyl-3-butene-2-ol	supercritical fluid extraction with CO_4_	calming (sedative) effects	[8,154]
	humulene	supercritical CO_2_ extraction with and without ultrasound assistance, temperatures (32–50 °C) and pressures (9.0–25.0 MPa)	mild corticosteroid effects	[107,155,156]
**Essential oils/polyfunctional thiols**	3-mercaptohexan-1-ol	pentane, dichloromethane, back-extraction of thiols from an organic solvent (pentane) to water	inhibition of EGCG oxidation	[145,157]

## Supplementary Materials

The following Figure S1 is available online at http://www.mdpi.com/2072-6643/11/2/257/s1, Figure S1. Chemical structures of all typical representatives of soft and hard resins are listed in Figure 3.

## Abbreviations

The following abbreviations are used in this manuscript.

3MH	3-mercaptohexan-1-ol
4MMP	4-methyl-4-mercaptopentan-2-one
8-PN	8-prenylnaringenin
BaP	benzo[a]pyrene
BMP7	bone morphogenetic protein 7
CB(1,2)	cannabinoid receptor 1, 2
CBMN	cytokinesis-block micronucleus
DPPH*	2,2-diphenyl-1-picrylhydrazyl radical
EBV-EA	Epstein–Barr virus early antigen
EGCG	epigallocatechin gallate
FGP	formamidopyrimidine DNA glycosylase
GC	gas chromatography
GC–MS	gas chromatography coupled with mass spectrometry
HDPE	high-density polyethylene
HPLC	high-performance liquid chromatography
IQ	2-amino-3-methyl-3H-imidazo[4,5-ƒ]quinolin
LCO_2_	liquid carbon dioxide
LDPE	low-density polyethylene
LTQ-Orbitrap-MS	ion trap quadrupole-Orbitrap-mass spectrometry
MAPK	mitogen-activated protein kinase
MHBA	matured hop bitter acids
MMP-9	matrix metalloproteinase
MNi	micronucleus index
MS/MS	two-stage mass analysis
MSn	multi-stage mass analysis
NBUDs	nuclear buds
NPBs	nucleoplasmic bridges
PCR	polymerase-chain-reaction
PE	polyethylene
PVPP	polyvinylpolypyrrolidone
ROS	reactive oxygen species
SCF	supercritical fluid
TPA	12-O-tetradecanoyl phorbol-13-acetat
XN	xanthohumol

## References

[1] Sawadogo W.R., Schumacher M., Teiten M.H., Dicato M., Diederich M. (2012). Traditional West African pharmacopeia, plants and derived compounds for cancer therapy. Biochem. Pharmacol..

[2] Labadie R.P., Van Der Nat J.M., Simons J.M., Kroes B.H., Kosasi S., Van Den Berg A.J., Hart L.A., Van Der Sluis W.G., Abeysekera A., Bamunuarachchi A. (1989). An ethnopharmacognostic approach to the search for immunomodulators of plant origin. Planta Med..

[3] Higdon J., Drake V.J. (2012). An Evidence-Based Approach to Phytochemicals and Other Dietary Factors.

[4] Zanoli P., Zavatti M. (2008). Pharmacognostic and pharmacological profile of *Humulus lupulus* L.. J. Ethnopharmacol..

[5] Prins S.J., Bates L.M., Keyes K.M., Muntaner C. (2015). Anxious? Depressed? You might be suffering from capitalism: Contradictory class locations and the prevalence of depression and anxiety in the United States. Sociol. Health Illn..

[6] Eyer J. (1980). Social Causes of Coronary Heart Disease. Psychother. Psychosom..

[7] Reganold J.P., Wachter J.M. (2016). Organic agriculture in the twenty-first century. Nat. Plants.

[8] Biendl M., Pinzl C. (2013). Hops and health Uses-Effects-History.

[9] Helmja K., Vaher M., Püssa T., Kamsol K., Orav A., Kaljurand M. (2007). Bioactive components of the hop strobilus: Comparison of different extraction methods by capillary electrophoretic and chromatographic methods. J. Chromatogr. A.

[10] Marriott J.R. (2010). Flavours-Greener chemistry preparation of traditional flavour extracts and molecules. Agro Food Ind. Hi-Tech.

[11] Daoud I.S., Kusinski S. (1986). Process aspects of the extraction of hops with liquid carbon dioxide. Inst. Brew..

[12] Green C.P., Osborne P. (1993). Rapid methods for obtaining essential oil from hops. J. Inst. Brew..

[13] Von Horst L.A.F., Kellner M. (1969). Thin Layer Steam Distillation of Hop Oil Extract. U.S. Patent.

[14] Pekhov A.V., Ponomaresko I.Y.A., Prokopchuk A.F. (1965). Thin Layer Steam Distillation of Hop Oil Extract. USSR Patent.

[15] Laws D.R.J., Bath N.A., Pickett J.A., Ennis C.S., Wheldon A.G. (1977). Preparation of Hop Extracts Without Using Organic Solvents. J. Inst. Brew..

[16] Muller A. Deutsche Auslegeschrift 2827002, 1978.

[17] Grant H.L. (1983). UK Patent.

[18] Manach C., Scalbert A., Morand C., Rémésy C., Jiménez L. (2004). Polyphenols: Food sources and bioavailability. Am. J. Clin. Nutr..

[19] He G.-Q., Xiong H.-P., Chen Q.-H., Ruan H., Wang Z.-Y., Traore L. (2005). Optimization of conditions for supercritical fluid extraction of flavonoids from hops (*Humulus lupulus* L.). J. Zhejiang Univ. Sci. B.

[20] Formato A., Gallo M., Ianniello D., Montesano D., Naviglio D. (2013). Supercritical fluid extraction of α and β acids from hops compared to cyclically pressurized solid–liquid extraction. J. Supercrit. Fluids.

[21] Stalikas C.D. (2007). Extraction, separation, and detection methods for phenolic acids and flavonoids. J. Sep. Sci..

[22] Ligor M., Stankevičius M., Wenda-Piesik A., Obelevičius K., Ragažinskienė O., Stanius Ž., Maruška A., Buszewski B. (2014). Comparative Gas Chromatographic–Mass Spectrometric Evaluation of Hop (*Humulus lupulus* L.) Essential Oils and Extracts Obtained Using Different Sample Preparation Methods. Food Anal. Methods.

[23] Sixt M., Koudous I., Strube J. (2016). Process design for integration of extraction, purification and formulation with alternative solvent concepts. Comptes Rendus Chim..

[24] Brglez Mojzer E., Knez-Hrnčič M., Škerget M., Knez Ž., Bren U. (2016). Polyphenols: Extraction methods, antioxidative action, bioavailability and anticarcinogenic effects. Molecules.

[25] De Vos D., Mertens P. (2011). United States Patent.

[26] Ochiai N., Sasamoto K., Kishimoto T. (2015). Development of a Method for the Quantitation of Three Thiols in Beer, Hop, and Wort Samples by Stir Bar Sorptive Extraction with in Situ Derivatization and Thermal Desorption–Gas Chromatography–Tandem Mass Spectrometry. J. Agric. Food Chem..

[27] Taniguchi Y., Matsukura Y., Taniguchi H., Koizumi H., Katayama M. (2015). Development of preparative and analytical methods of the hop bitter acid oxide fraction and chemical properties of its components. Biosci. Biotechnol. Biochem..

[28] Sohrabvandi S., Mortazavian A.M., Rezaei K. (2011). Advanced Analytical Methods For The Analysis of Chemical And Microbiological Properties of Beer. J. Food Drug Anal..

[29] Quifer-Rada P., Vallverdú-Queralt A., Martínez-Huélamo M., Chiva-Blanch G., Jáuregui O., Estruch R., Lamuela-Raventós R. (2015). A comprehensive characterisation of beer polyphenols by high resolution mass spectrometry (LC–ESI-LTQ-Orbitrap-MS). Food Chem..

[30] Stevens J.F., Page J.E. (2004). Xanthohumol and related prenylflavonoids from hops and beer: To your good health!. Phytochemistry.

[31] Paunović D.Đ., Mitić S.S., Stojanović G.S., Mitić M.N., Stojanović B.T., Stojković M.B. (2015). Kinetics of the Solid-Liquid Extraction Process of Phenolic Antioxidants and Antioxidant Capacity from Hop (*Humulus lupulus* L.. Separ. Sci. Technol..

[32] Barbosa-Pereira L., Bilbao A., Vilches P., Angulo I., Lluis J., Fité B., Paseiro-Losada P., Cruz J.M. (2014). Brewery waste as a potential source of phenolic compounds: Optimisation of the extraction process and evaluation of antioxidant and antimicrobial activities. Food Chem..

[33] Abram V., Čeh B., Vidmar M., Hercezi M., Lazić N., Bucik V., Smole Mozina S., Kosir I.J., Kac M., Demšar L. (2015). A comparison of antioxidant and antimicrobial activity between hop leaves and hop cones. Ind. Crop. Prod..

[34] Spreng S., Hofmann T. (2018). Activity-Guided Identification of in Vitro Antioxidants in Beer. J. Agric. Food Chem..

[35] Teuber M., Schmalreck A.F. (1973). Membrane Leakage in Bacillus subtilis 168 induced by the hop constituents lupulone, humulone, isohumulone and humulinic acid. Arch. Microbiol..

[36] Dušek M., Jandovská V., Čermák P., Mikyška A., Olšovská J. (2016). A novel approach for identification of biologically active phenolic compounds in complex matrices using hybrid quadrupole-orbitrap mass spectrometer: A promising tool for testing antimicrobial activity of hops. Talanta.

[37] Bartmańska A., Wałecka-Zacharska E., Tronina T., Popłoński J., Sordon S., Brzezowska E., Bania J., Huszcza E. (2018). Antimicrobial Properties of Spent Hops Extracts, Flavonoids Isolated Therefrom, and Their Derivatives. Molecules.

[38] Rauha J.-P., Remes S., Heinonen M., Hopia A., Kähkönen M., Kujala T., Pihlaja K., Vuorela H., Vuorela P. (2000). Antimicrobial effects of Finnish plant extracts containing flavonoids and other phenolic compounds. Int. J. Food Microbiol..

[39] Groppo F.C., Bergamaschi C.D.C., Cogo K., Franz-Montan M., Motta R.H.L., De Andrade E.D., Cogo-Muller K., Franz-Montan M. (2008). Use of phytotherapy in dentistry. Phytother. Res..

[40] Shelef L.A., Naglik O.A., Bogen D.W. (1980). Sensitivity of some common food-borne bacteria to the spices sage, rosemary, and allspice. J. Food Sci..

[41] Tajkarimi M.M., Ibrahim S.A., Cliver D.O. (2010). Antimicrobial herb and spice compounds in food. Food Control.

[42] Vaughan A., O’Sullivan T., van Sinderen D. (2005). Enhancing the microbiological stability of malt and beer—A review. J. Inst. Brew..

[43] Puupponen-Pimiä R., Nohynek L., Meier C., Kähkönen M., Heinonen M., Hopia A. (2001). Antimicrobial properties of phenolic compounds from berries. J. Appl. Microbiol..

[44] Brantner A., Grein E. (1994). Antibacterial activity of plant extracts used externally in traditional medicine. J. Ethnopharmacol..

[45] Panche A.N., Diwan A.D., Chandra S.R. (2016). Flavonoids: An overview. J. Nutr. Sci..

[46] Spanou C., Bourou G., Dervishi A., Aligiannis N., Angelis A., Komiotis D., Skaltsounis A.L., Kouretas D. (2008). Antioxidant and Chemopreventive Properties of Polyphenolic Compounds Derived from Greek Legume Plant Extracts. J. Agric. Food Chem..

[47] Rój E., Tadić V.M., Mišić D., Žižović I., Arsić I., Dobrzyńska-Inger A., Kostrzewa D. (2015). Supercritical carbon dioxide hops extracts with antimicrobial properties. Open Chem..

[48] Di Sotto A., Di Giacomo S., Abete L., Božović M., Parisi O.A., Barile F., Vitalone A., Izzo A.A., Ragno R., Mazzanti G. (2017). Genotoxicity assessment of piperitenone oxide: An in vitro and in silico evaluation. Food Chem. Toxicol..

[49] Plazar J. (2007). Mechanism of Antigenotoxic Activity of Xanthohumol and Related Prenylflavonoids from Hops (*Humulus lupulus* L.). Dissertation Thesis.

[50] Hennig B., Petriello M.C., Gamble M.V., Surh Y.J., Kresty L.A., Frank N., Rangkadilok N., Ruchirawat M., Suk W.A. (2018). The role of nutrition in influencing mechanisms involved in environmentally mediated diseases. Rev. Environ. Health.

[51] Collins A.R. (2014). Measuring oxidative damage to DNA and its repair with the comet assay. Biochim. Biophys. Acta.

[52] Collins A., Koppen G., Valdiglesias V., Dusinska M., Kruszewski M., Møller P., Rojas E., Dhawan A., Benzie I., Coskun E. (2014). The comet assay as a tool for human biomonitoring studies: The ComNet Project. Mutat. Res..

[53] Staruchova M., Collins A.R., Volkovova K., Mislanová C., Kovacikova Z., Tulinska J., Kocan A., Staruch L., Wsolova L., Dusinska M. (2008). Occupational exposure to mineral fibres. Biomarkers of oxidative damage and antioxidant defence and associations with DNA damage and repair. Mutagenesis.

[54] Collins A.R., Azqueta A. (2012). DNA repair as a biomarker in human biomonitoring studies; further applications of the comet assay. Mutat. Res..

[55] Fenech M. (2006). Cytokinesis-block micronucleus assay evolves into a “cytome” assay of chromosomal instability, mitotic dysfunction and cell death. Mutat. Res..

[56] Novak M., Žegura B., Modic B., Heath E., Filipič M. (2017). Cytotoxicity and genotoxicity of anticancer drug residues and their mixtures in experimental model with zebrafish liver cells. Sci. Total Environ..

[57] Neiens S.D., Steinhaus M. (2018). Odor-Active Compounds in the Special Flavor Hops Huell Melon and Polaris. J. Agric. Food Chem..

[58] Štulíková K., Karabín M., Nešpor J., Dostálek P. (2018). Therapeutic Perspectives of 8-Prenylnaringenin, a Potent Phytoestrogen from Hops. Molecules.

[59] Mutlu Altundağ E., Yılmaz A.M., Koçtürk S., Taga Y., Yalçın A.S. (2018). Synergistic Induction of Apoptosis by Quercetin and Curcumin in Chronic Myeloid Leukemia (K562) Cells. Nutr. Cancer.

[60] Steenackers B., De Cooman L., De Vos D. (2015). Chemical transformations of characteristic hop secondary metabolites in relation to beer properties and the brewing process: A review. Food Chem..

[61] Van Cleemput M., Cattoor K., De Bosscher K., Haegeman G., De Keukeleire D., Heyerick A. (2009). Hop (*Humulus lupulus*)-Derived Bitter Acids as Multipotent Bioactive Compounds. J. Nat. Prod..

[62] Pichler C., Ferk F., Al-Serori H., Huber W., Jäger W., Waldherr M., Mišík M., Kundi M., Nersesyan A., Herbacek I. (2017). Xanthohumol Prevents DNA Damage by Dietary Carcinogens: Results of a Human Intervention Trial. Cancer Prev. Res..

[63] Niedzwiecki A., Roomi M.W., Kalinovsky T., Rath M. (2016). Anticancer Efficacy of Polyphenols and Their Combinations. Nutrients.

[64] Zhou Y., Zheng J., Li Y., Xu D.P., Li S., Chen Y.M., Li H.B. (2016). Natural Polyphenols for Prevention and Treatment of Cancer. Nutrients.

[65] Jiang J., Xong Y.L. (2016). Natural antioxidants as food and feed additives to promote health benefits and quality of meat products: A review. Meat Sci..

[66] Villalobos-Delgado L.H., Caro I., Blanco C., Bodas R., Andrés S., Giráldez F.J., Mateo J. (2015). Effect of the addition of hop (infusion or powder) on the oxidative stability of lean lamb patties during storage. Small Ruminant Res..

[67] De Keukeleire J., Ooms G., Heyerick A., Roldan-Ruiz I., Van Bockstaele E., De Keukeleire D. (2003). Formation and accumulation of alpha-acids, beta-acids, desmethylxanthohumol, and xanthohumol during flowering of hops (*Humulus lupulus* L.). J. Agric. Food Chem..

[68] Okada Y., Ito K. (2001). Cloning and analysis of valerophenone synthase gene expressed specifically in lupulin gland of hop (*Humulus lupulus* L.). Biosci. Biotechnol. Biochem..

[69] Chadwick L.R., Pauli G.F., Farnsworth N.R. (2006). The pharmacognosy of *Humulus lupulus* L. (hops) with an emphasis on estrogenic properties. Phytomedicine.

[70] Lamy V., Roussi S., Chaabi M., Gossé F., Schall N., Lobstein A., Raul F. (2007). Chemopreventive effects of lupulone, a hop β-acid, on human colon cancer-derived metastatic SW620 cells and in a rat model of colon carcinogenesis. Carcinogenesis.

[71] Matsui H., Inui T., Oka K., Fukui N. (2016). The influence of pruning and harvest timing on hop aroma, cone appearance, and yield. Food Chem..

[72] Schurr B.C., Hahne H., Kuster B., Behr J., Vogel R.F. (2015). Molecular mechanisms behind the antimicrobial activity of hop iso-a-acids in Lactobacillus brevis. Food Microbiol..

[73] Kurasawa T., Chikaraishi Y., Naito A., Toyoda Y., Notsu Y. (2005). Effect of *Humulus lupulus* on Gastric Secretion in Rat Pylorus-Ligated Model. Biol. Pharm. Bull..

[74] Walker J., Hell J., Liszt K.I., Dresel M., Pignitter M., Hofmann T., Somoza V. (2012). Identification of Beer Bitter Acids Regulating Mechanisms of Gastric Acid Secretion. J. Agric. Food Chem..

[75] Schiller H., Forster A., Vonhoff C., Hegger M., Biller A., Winterhoff H. (2006). Sedating effects of *Humulus lupulus* L. extracts. Phytomedicine.

[76] Bortoluzzi C., Menten J.F.M., Silveira H., Melo A.D.B., Rostagno M.H. (2016). Hops β-acids (*Humulus lupulus*) decrease intestinal gene expression of proinflammatory cytokines in an ex-vivo model. J. Appl. Poult. Res..

[77] Sandoval-Ramírez B.A., Lamuela-Raventós R.M., Estruch R., Sasot G., Doménech M., Tresserra-Rimbau A. (2017). Beer Polyphenols and Menopause: Effects and Mechanisms-A Review of Current Knowledge. Oxid. Med. Cell Longev..

[78] Chen W.J., Lin J.K. (2004). Mechanisms of Cancer Chemoprevention by Hop Bitter Acids (Beer Aroma) through Induction of Apoptosis Mediated by Fas and Caspase Cascades. J. Agric. Food Chem..

[79] Lamy V., Roussi S., Chaabi M., Gossé F., Lobstein A., Raul F. (2008). Lupulone, a hop bitter acid, activates different death pathways involving apoptotic TRAIL-receptors, in human colon tumor cells and in their derived metastatic cells. Apoptosis.

[80] Lamy V., Bousserouel S., Gossé F., Minker C., Lobstein A., Raul F. (2011). Lupulone triggers p38 MAPK-controlled activation of p53 and of the TRAIL receptor apoptotic pathway in human colon cancer-derived metastatic cells. Oncol. Rep..

[81] Saugspier M., Dorn C., Czech B., Gehrig M., Heilmann J., Hellerbrand C. (2012). Hop bitter acids inhibit tumorigenicity of hepatocellular carcinoma cells in vitro. Oncol. Rep..

[82] Karabín M., Hudcová T., Jelínek L., Dostálek P. (2016). Biologically Active Compounds from Hops and Prospects for Their Use. Compr. Rev. Food Sci. Food Saf..

[83] Hougee S., Faber J., Sanders A., van den Berg W.B., Garssen J., Smit H.F., Hoijer M.A. (2006). Selective inhibition of COX-2 by a standardized CO 2 extract of *Humulus lupulus* in vitro and its activity in a mouse model of zymosan-induced arthritis. Planta Med..

[84] Lee J.C., Kundu J.K., Hwang D.M., Na H.K., Surh Y.J. (2007). Humulone inhibits phorbol ester-induced COX-2 expression in mouse skin by blocking activation of NF-kappa B and AP-1: I kappa B kinase and c-Jun-N-terminal kinase as respective potential upstream targets. Carcinogenesis.

[85] Hsu C.H., Ho Y.S., Lai C.S., Hsieh S.C., Chen L.H., Lin E., Ho C.T., Pan M.H. (2013). Hexahydro-β-acids potently inhibit 12-O-tetradecanoylphorbol 13-acetate-induced skin inflammation and tumor promotion in mice. J. Agric. Food Chem..

[86] Akazawa H., Kohno H., Tokuda H., Suzuki N., Yasukawa K., Kimura Y., Manosroi A., Manosroi J., Akihisa T. (2012). Anti-Inflammatory and Anti-Tumor-Promoting Effects of 5-Deprenyllupulonol C and Other Compounds from Hop (*Humulus lupulus* L.). Chem. Biodivers..

[87] Menezes J.C., Orlikova B., Morceau F., Diederich M. (2016). Natural and Synthetic Flavonoids: Structure Activity Relationship and Chemotherapeutic Potential for the Treatment of Leukemia. Crit. Rev. Food Sci. Nutr..

[88] Terao J., Mukai R. (2014). Prenylation modulates the bioavailability and bioaccumulation of dietary flavonoids. Arch. Biochem. Biophys..

[89] Legette I., Ma L., Reed R.L., Miranda C.L., Christensen J.M., Rodriguez-Proteau R., Stevens J.F. (2012). Pharmacokinetics of xanthohumol and metabolites in rats after oral and intravenous administration. Mol. Nutr. Food Res..

[90] Cattoor K., Bracke D., Deforce D., de Keukeleire D., Heyerick A. (2010). Transport of hop bitter acids across intestinal Caco-2 cell monolayers. J. Agric. Food Chem..

[91] Konishi Y., Hitomi Y., Yodhida M., Yoshida E. (2005). Absorption and bioavailability of artepillin C in rats after oral administration. J. Agric. Food Chem..

[92] Mukai R., Horikawa H., Fujikura Y., Kawamura T., Nemoto H., Nikawa T., Terao J. (2012). Prevention of disuse muscle atrophy by dietary ingestion of 8-prenylnaringenin in denervated mice. PLoS ONE.

[93] Mukai R., Fujikura Y., Murota K., Uehara M., Minekawa S., Matsui N., Kawamura T., Nemoto H., Terao J. (2013). Prenylation enhances quercetin uptake and reduces efflux in Caco-2 cells and enhances tissue accumulation in mice fed long-term. J. Nutr..

[94] Calvo-Castro L.A., Burkard M., Sus N., Scheubeck G., Leischner C., Lauer U.M., Bosy-Westphal A., Hund V., Busch C., Venturelli S. (2018). The Oral Bioavailability of 8-Prenylnaringenin from Hops (*Humulus lupulus* L.) in Healthy Women and Men is Significantly Higher than that of its Positional Isomer 6-Prenylnaringenin in a Randomized Crossover Trial. Mol. Nutr. Food Res..

[95] Gerhäuser C., Alt A., Heiss E., Gamal-Eldeen A., Klimo K., Knauft J., Neumann I., Scherf H.R., Frank N., Bartsch H. (2002). Cancer chemopreventive activity of Xanthohumol, a natural product derived from hop. Mol. Cancer Ther..

[96] Miranda C.L., Aponso G.I., Stevens J.F., Deinzer M.L., Buhler D.R. (2000). Prenylated chalcones and flavanones as inducers of quinone reductase in mouse Hepa 1c1c7 cells. Cancer Lett..

[97] Dietz B.M., Kang Y.H., Liu G., Eggler A.L., Yao P., Chadwick L.R., Pauli G.F., Farnsworth N.R., Mesecar A.D., van Breemen R.B. (2005). Xanthohumol isolated from *Humulus lupulus* inhibits menadione-induced DNA damage through induction of quinone reductase. Chem. Res. Toxicol..

[98] Gerhäuser C., Preedy V.R. (2009). Phenolic beer compounds to prevent cancer. Beer in Health and Disease Prevention.

[99] Harikumar K.B., Kunnumakkara A.B., Ahn K.S., Anand P., Krishnan S., Guha S., Aggarwa B.B. (2009). Modification of the cysteine residues in IkB kinase and NF-kB (p65) by xanthohumol leads to suppression of NF-kB–regulated gene products and potentiation of apoptosis in leukemia cells. Blood.

[100] Pan L., Becker H., Gerhäuser C. (2005). Xanthohumol induces apoptosis in cultured 40-16 human colon cancer cells by activation of the death receptor- and mitochondrial pathway. Mol. Nutr. Food Res..

[101] Yang J.-Y., Della-Fera M.A., Rayalam S., Baile C.A. (2007). Effect of xanthohumol and isoxanthohumol on 3T3-L1 cell apoptosis and adipogenesis. Apoptosis.

[102] Zhang B., Chu W., Wei P., Liu Y., Wei T. (2015). Xanthohumol induces generation of reactive oxygen species and triggers apoptosis through inhibition of mitochondrial electron transfer chain complex I. Free Radic. Biol. Med..

[103] Mojzis J., Varinska L., Mojzisova G., Kostova I., Mirossay L. (2008). Antiangiogenic effects of flavonoids and chalcones. Pharmacol. Res..

[104] Stracke D., Schulz T., Prehm P. (2011). Inhibitors of hyaluronan export from hops prevent osteoarthritic reactions. Mol. Nutr. Food Res..

[105] Nozawa H., Nakao W., Zhao F., Kondo K. (2005). Dietary supplement of isohumulones inhibits the formation of aberrrant crypt foci with a concomitant decrease in prostaglandin E2 level in rat colon. Mol. Nutr. Food Res..

[106] Rudzitis-Auth J., Körbel C., Scheuer C., Menger M.D., Laschke M.W. (2012). Xanthohumol inhibits growth and vascularization of developing endometriotic lesions. Hum. Reprod..

[107] Ameh S.J., Ibekwe N.N., Ebeshi B.U. (2015). Essential Oils in Ginger, Hops, Cloves, and Pepper Flavored Beverages–A Review. J. Dietary Suppl..

[108] Rad M., Hümpel M., Schaefer O., Schoemaker R.C., Schleuning W.D., Cohen A.F., Burggraaf J. (2006). Pharmacokinetics and systemic endocrine effects of the phyto-oestrogen 8-prenylnaringenin after single oral doses to postmenopausal women. Br. J. Clin. Pharmacol..

[109] Keiler A.M., Macejova D., Dietz B.M., Bolton J.L., Pauli G.F., Chen S.N., van Breemen R.B., Nikolic D., Goerl F., Muders M.H. (2017). Evaluation of estrogenic potency of a standardized hops extract on mammary gland biology and on MNU-induced mammary tumor growth in rats. J. Steroid. Biochem. Mol. Biol..

[110] Erkkola R., Vervarcke S., Vansteelandt S., Rompotti P., De Keukeleire D., Heyerick A. (2010). A randomized, double-blind, placebo-controlled, cross-over pilot study on the use of a standardized hop extract to alleviate menopausal discomforts. Phytomedicine.

[111] Brunelli E., Minassi A., Appendino G., Moro L. (2007). 8-Prenylnaringenin, inhibits estrogen receptor-alfa mediated cell growth and induces apoptosis in MCF-7 breast cancer cells. J. Steroid Biochem. Mol. Biol..

[112] Delmulle L., Vanden Berghe T., De Keukeleire D., Vandenabeele P. (2008). Treatment of PC-3 and DU145 Prostate Cancer Cells by Prenylflavonoids from Hop (*Humulus lupulus* L.) induces a Caspase-independent Form of Cell Death. Phytother. Res..

[113] Diller R.A., Riepl H.M., Rose O., Frias C., Henze G., Prokop A. (2005). Synthesis of Demethylxanthohumol, a New Potent Apoptosis-Inducing Agent from Hops. Chem. Biodivers..

[114] Gerhauser C. (2005). Broad spectrum antiinfective potential of xanthohumol from hop (*Humulus lupulus* L.) in comparison with activities of other hop constituents and xanthohumol metabolites. Mol. Nutr. Food Res..

[115] Fresco P., Borges F., Diniz C., Marques M.P.M. (2006). New Insights on the Anticancer Properties of Dietary Polyphenols. Med. Res. Rev..

[116] Kampa M., Nifli A.-P., Notas G., Castanas E. (2007). Polyphenols and cancer cell growth. Rev. Physiol. Biochem. Pharmacol..

[117] Corrêa R.C.G., Peralta R.M., Haminiuk C.W.I., Maciel G.M., Bracht A., Ferreira I.C.F.R. (2018). New phytochemicals as potential human anti-aging compounds: Reality, promise, and challenges. Crit. Rev. Food Sci. Nutr..

[118] Sharmila G., Bhat F.A., Arunkumar R., Elumalai P., Singh P.R., Senthilkumar K., Arunakaran J. (2014). Chemopreventive effect of quercetin, a natural dietary flavonoid on prostate cancer in in vivo model. Clin. Nutr..

[119] Tabrez S.T., Priyadarshini M., Urooj M., Shakil S., Ashraf G.M., Khan M.S., Kamal M.A., Alam Q., Jabir N.R., Abuzenadah A.M. (2013). Cancer Chemoprevention by Polyphenols and Their Potential Application as Nanomedicine. J. Environ. Sci. Health Part C.

[120] Lewandowska H., Kalinowska M., Lewandowski W., Stępkowski T.M., Brzóska K. (2015). The role of natural polyphenols in cell signaling and cytoprotection against cancer development. J. Nutr. Biochem..

[121] Spagnuolo C., Russo M., Bilotto S., Tedesco I., Laratta B., Russo G.L. (2012). Dietary polyphenols in cancer prevention: The example of the flavonoid quercetin in leukemia. Ann. N. Y. Acad. Sci..

[122] Dai J., Mumper J.R. (2010). Plant Phenolics: Extraction, Analysis and Their Antioxidant and Anticancer Properties. Molecules.

[123] Segawa S., Yasui K., Takata Y., Kurihara T., Kaneda H., Watari J. (2006). Flavonoid Glycosides Extracted from Hop (*Humulus lupulus* L.) as Inhibitors of Chemical Mediator Release from Human Basophilic KU812 Cells. Biosci. Biotechnol. Biochem..

[124] Kosasi S., Van Der Sluism W.G., Labadie R.P. (1989). Multifidol and Multifidol Glucoside From the Latex of Jatropha Multifida. Phytochemistry.

[125] Bohr G., Gerhäuser C., Knauft J., Zapp J., Becker H. (2005). Anti-inflammatory Acylphloroglucinol Derivatives from Hops (*Humulus lupulus*). J. Nat. Prod..

[126] Lu D.-Y., Chang C.-S., Yeh W.-L., Tang C.-H., Cheung C.-W., Leung Y.-M., Liu J.-F., Wong K.-L. (2012). The novel phloroglucinol derivative BFP induces apoptosis of glioma cancer through reactive oxygen species and endoplasmic reticulum stress pathways. Phytomedicine.

[127] Dresel M., Dunkel A., Hofmann T. (2015). Sensomics Analysis of Key Bitter Compounds in the Hard Resin of Hops (*Humulus lupulus* L.) and Their Contribution to the Bitter Profile of Pilsner-Type Beer. J. Agric. Food Chem..

[128] Ghosh S., Basak P., Duttam S., Chowdhury S., Sil P.C. (2017). New insights into the ameliorative effects of ferulic acid in pathophysiological conditions. Food Chem. Toxicol..

[129] Zhang X., Lin D., Jiang R., Li H., Wan J., Li H. (2016). Ferulic acid exerts antitumor activity and inhibits metastasis in breast cancer cells by regulating epithelial to mesenchymal transition. Oncol. Rep..

[130] Yang C.S., Landau J.M., Huang M.-T., Newmark H.L. (2001). Inhibition of carcinogenesis by dietary Polyphenolic compounds. Annu. Rev. Nutr..

[131] Izquierdo-Torres E., Rodríguez G., Meneses-Morales I., Zarain-Herzberg A. (2017). ATP2A3 gene as an important player for resveratrol anticancer activity in breast cancer cells. Mol. Carcinog..

[132] Zeng Y.H., Zhou L.Y., Chen Q.Z., Li Y., Shao Y., Ren W.Y., Liao Y.P., Wang H., Zhu J.H., Huang M. (2017). Resveratrol inactivates PI3K/Akt signaling through upregulating BMP7 in human colon cancer cells. Oncol. Rep..

[133] Park E.J., Pezzuto J.M. (2015). The pharmacology of resveratrol in animals and humans. Biochim. Biophys. Acta.

[134] Roland A., Viel C., Reillon F., Delpech S., Boivin P., Schneider R., Dagan L. (2016). First identification and quantification of glutathionylated and cysteinylated precursors of 3-mercaptohexan-1-ol and 4-methyl-4-mercaptopentan-2-one in hops (*Humulus lupulus*). Flavour. Fragr. J..

[135] Kaškonas P., Stanius Ž., Kaškonienė V., Obelevičius K., Ragažinskienė O., Žilinskas A., Maruška A. (2016). Clustering analysis of different hop varieties according to their essential oil composition measured by GC/MS. Chem. Pap..

[136] Okaru A.O., Lachenmeier D.W. (2017). The Food and Beverage Occurrence of Furfuryl Alcohol and Myrcene—Two Emerging Potential Human Carcinogens?. Toxics.

[137] Mitić-Ćulafić D., Žegura B., Filipič M., Nikolić B., Jovanović M., Knežević-Vukčević J. (2016). Antigenotoxic potential of plant monoterpenes linalool, myrcene and eucalyptol against IQ- and PhIP- induced DNA damage. Botanica Serbica.

[138] Lee J.-H., Lee K., Lee D.H., Shin S.Y., Yong Y., Lee Y.H. (2015). Anti-invasive effect of β-myrcene, a component of the essential oil from Pinus koraiensis cones, in metastatic MDA-MB-231 human breast cancer cells. J. Korean Soc. Appl. Biol. Chem..

[139] Bedini S., Flamini G., Girardi J., Cosci F., Conti B. (2015). Not just for beer: Evaluation of spent hops (*Humulus lupulus* L.) as a source of eco-friendly repellents for insect pests of stored foods. J. Pest. Sci..

[140] Zhang Z., Guo S., Liu X., Gao X. (2015). Synergistic Antitumor Effect of α-pinene and β-pinene with Paclitaxel against Non-small-cell Lung Carcinoma (NSCLC). Drug. Res..

[141] Fidyt K., Fiedorowicz A., Strządała L., Szumny A. (2016). β-caryophyllene and β-caryophyllene oxide—Natural compounds of anticancer and analgesic properties. Cancer Med..

[142] Peppard T.L. (1981). Volatile Organosulphur Compounds in Hops and Hop Oils: A Review. J. Inst. Brew..

[143] Guadagni D.G., Buttery R.G., Harris J. (1966). Odour intensities of hop oil components. J. Sci. Food Agric..

[144] Cibaka M.-L.K., Decourrière L., Lorenzo-Alonso C.-J., Bodart E., Robiette R., Collin S. (2016). 3-Sulfanyl-4-methylpentan-1-ol in Dry-Hopped Beers: First Evidence of Glutathione S-Conjugates in Hop (*Humulus lupulus* L.). J. Agric. Food Chem..

[145] Unnadkat N.R., Elias R.J. (2012). Oxidative Stability of (-)-Epigallocatechin Gallate in the Presence of Thiols. J. Agric. Food Chem..

[146] Yun J.-M., Lian W.-Q., Pu L.-M., Xue H.-L., Ai D.-Y., Zhang W.-W. (2012). Optimization of Extraction Process for Humulone and Lupulone from Hop Extract by Acid-Alkali Precipitation. Food Sci..

[147] Zekovic Z., Pfaf-Sovljanski I., Grujic O. (2007). Supercritical Fluid Extraction of Hops. J. Serb. Chem. Soc..

[148] Hudcová T., Bryndová J., Fialová K., Fiala J., Karabín M., Jelínek L., Dostálek P. (2014). Antiproliferative effects of prenylflavonoids from hops on human colon cancer cell lines. J. Inst. Brew..

[149] Gil-Ramírez A., Mendiola J.A., Arranz E., Ruíz-Rodríguez A., Reglero G., Ibáñez E., Marín F.R. (2012). Highly isoxanthohumol enriched hop extract obtained by pressurized hot water extraction (PHWE). Chemical and functional characterization. Innov. Food Sci. Emerg..

[150] Perva-Uzunalic A., Škerget M., Knez Ž., Weinreich B., Otto F., Gruner S. (2006). Extraction of active ingredients from green tea (Camellia sinensis): Extraction efficiency of major catechins and caffeine. Food Chem..

[151] Kishimoto T., Wanikawa A., Kagami N., Kawatsura K. (2000). Analysis of hop-derived terpenoids in beer and evaluation of their behaviour using the stir bar sorptive extraction method with GC-MS. J. Agric. Food Chem..

[152] Buranov A.U., Mazza G. (2009). Extraction and purification of ferulic acid from flax shives, wheat and corn bran by alkaline hydrolysis and pressurised solvents. Food Chem..

[153] Soural I., Vrchotová N., Tříska J., Balík J., Horník Š., Cuřínová P., Sýkora J. (2015). Various extraction methods for obtaining stilbenes from grape cane of Vitis vinifera L.. Molecules.

[154] Lemberkovics E., Kéry A., Simándi B., Kakasy A., Balázs A., Héthelyi E., Szoke E. (2004). Influence of extraction methods on the composition of essential oils. Acta Pharm. Hung..

[155] Wei M.C., Xiao J., Yang Y.C. (2016). Extraction of α-humulene-enriched oil from clove using ultrasound-assisted supercritical carbon dioxide extraction and studies of its fictitious solubility. Food Chem..

[156] Vinatoru M., Toma M., Radu O., Filip P.I., Lazurca D., Mason T.J. (1997). The use of ultrasound for the extraction of bioactive principles from plant materials. Ultrason. Sonochem..

[157] Piano F., Fracassetti D., Buica A., Stander M., du Toit W.J., Borsa D., Tirelli A. (2015). Development of a novel liquid/liquid extraction and ultra-performance liquid chromatography tandem mass spectrometry method for the assessment of thiols in South African Sauvignon Blanc wines. Aust. J. Grape Wine R..

[158] Russ W., Meyer-Pittroff R. (2004). Utilizing waste products from food production and processing industries. Crit. Rev. Food Sci..

[159] Mastanjević K., Krstanović V., Lukinac J., Jukić M., Vulin Z., Mastanjević K. (2018). Beer–The Importance of Colloidal Stability (Non-Biological Haze). Fermentation.

[160] Wietstock P.C., Glattfelder R., Garbe L.A., Methner F.J. (2016). Characterization of the Migration of Hop Volatiles into Different Crown Cork Liner Polymers. J. Agric. Food. Chem..

[161] Santos I.S., Ponte B.M., Boonme P., Silva A.M., Souto E.B. (2013). Nanoencapsulation of polyphenols for protective effect against colon–rectal cancer. Biotechnol. Adv..

[162] Siddiqui I.A., Sanna V., Ahmad N., Sechi M., Mukhtar H. (2015). Resveratrol nanoformulation for cancer prevention and therapy. Ann. N.Y. Acad. Sci..

[163] Murota K., Matsuda N., Kashino Y., Fujikura Y., Nakamura T., Kato Y., Shimizu R., Okuyam S., Tanaka H., Koda T. (2010). alpha-Oligoglucosylation of a sugar moiety enhances the bioavailability of quercetin glucosides in humans. Arch. Biochem. Biophys..

[164] Takumi H., Nakamura H., Simizu T., Harada R., Kometani T., Nadamoto T., Mukai R., Murota K.Y., Kawai Y., Terao J. (2012). Bioavailability of orally administered water-dispersible hesperetin and its effect on peripheral vasodilatation in human subjects: Implication of endothelial functions of plasma conjugated metabolites. Food Funct..

[165] Knez Ž., Knez Hrnčič M., Škerget M. (2015). Particle Formation and Product Formulation Using Supercritical Fluids. Annu. Rev. Chem. Biomol. Eng..

[166] Lajovic A., Nagy L.D., Guengerich F.P., Bren U. (2015). Carcinogenesis of urethane: Simulation versus experiment. Chem. Res. Toxicol..

[167] Brown K.L., Bren U., Stone M.P., Guengerich F.P. (2009). Inherent stereospecificity in the reaction of aflatoxin B1 8, 9-epoxide with deoxyguanosine and efficiency of DNA catalysis. Chem. Res. Toxicol..

[168] Galeša K., Bren U., Kranjc A., Mavri J. (2008). Carcinogenicity of acrylamide: A computational study. J. Agric. Food Chem..

[169] Gaussian.com Expanding the Limits of Computational Chemistry. http://gaussian.com/.

[170] MOLDEN a Visualization Program of Molecular and Electronic structure. http://cheminf.cmbi.ru.nl/molden/.

[171] Gladović M., Španinger E., Bren U. (2018). Nucleic bases alkylation with acrylonitrile and cyanoethylene oxide: A computational study. Chem. Res. Toxicol..

[172] Frisch M.J., Trucks G.W., Schlegel H.B., Scuseria G.E., Robb M.A., Cheeseman J.R., Scalmani G., Barone V., Mennucci B., Petersson G.A. (2009). Gaussian 09.

