# Supplementary material for: Hop Compounds: Extraction Techniques, Chemical Analyses, Antioxidative, Antimicrobial, and Anticarcinogenic Effects
Source: Nutrients. 2019 Jan 24;11(2):257. doi: 10.3390/nu11020257 (PMC6412513; doi:10.3390/nu11020257)
Supplement: Supplementary file 1 [file nutrients-11-00257-s001.pdf]

Supplementary material

Hop compounds: extraction techniques, chemical analyses, antioxidative, antimicrobial and anticarcinogenic effects

Maša Knez Hrnčič [1,#], Eva Španinger [2,#], Iztok Jože Košir [3], Željko Knez [1] and Urban Bren [2,*]

[1] Laboratory of Separation Processes and Product Design, Faculty of Chemistry and Chemical Engineering, University of Maribor, Smetanova ulica 17, SI-2000 Maribor, Slovenia.
[2] Laboratory of Physical Chemistry and Chemical Thermodynamics, Faculty of Chemistry and Chemical Engineering, University of Maribor, Smetanova ulica 17, SI-2000 Maribor, Slovenia.
[3] Slovenian Institute of Hop Research and Brewing, Cesta Žalskega Tabora 2, SI-3310 Žalec, Slovenia.
\# These authors contributed equally to this work.
* Correspondence: urban.bren@um.si; Tel.: +386-2-2294-421

Figure S1. Chemical structures of typical representatives of soft and hard resins.

Class	Structure	Representatives
**Soft resins/ Bitter acids**<br><br>Alpha-acids and derivatives	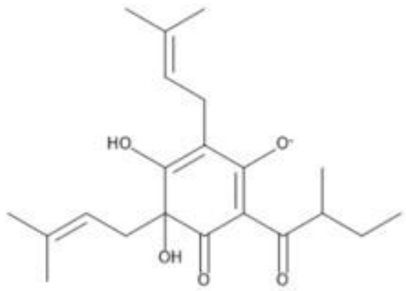	Adhumulone
	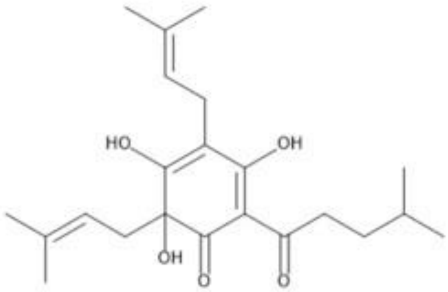	Adprehumolone
	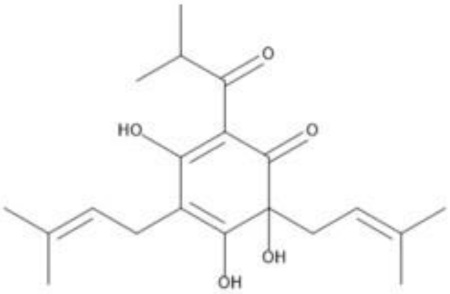	Cohumolone

**Soft resins/ Bitter acids**<br><br>Alpha-acids and derivatives	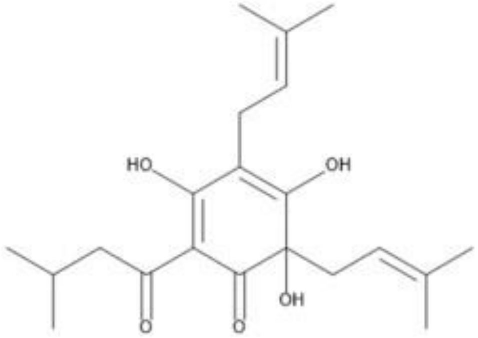	Humulone
	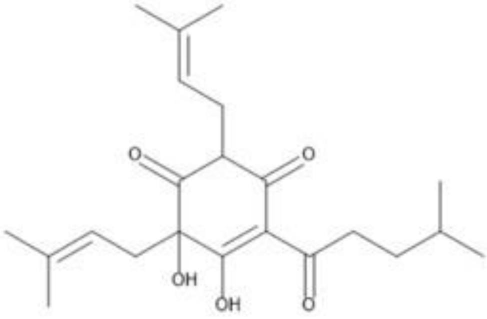	Prehumulone
	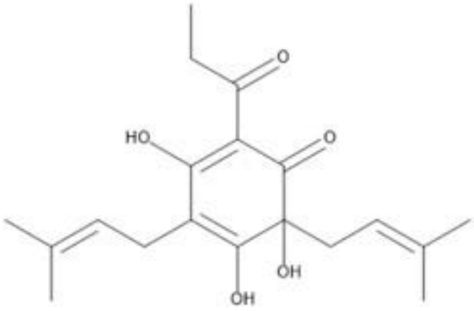	Posthumolone
	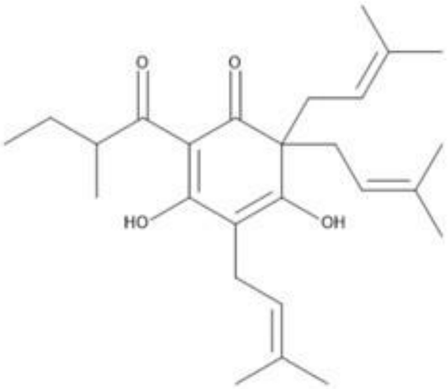	Adlupulone

**Soft resins/ Bitter acids**<br><br>Beta-acids and derivatives	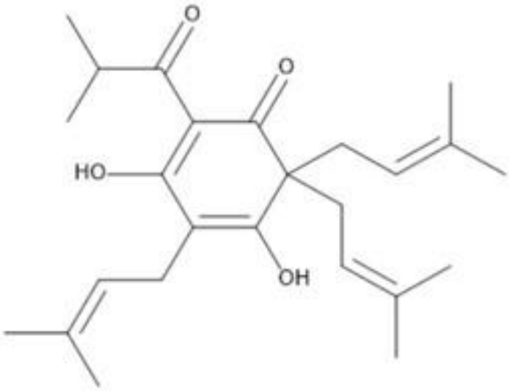	Colupulone
	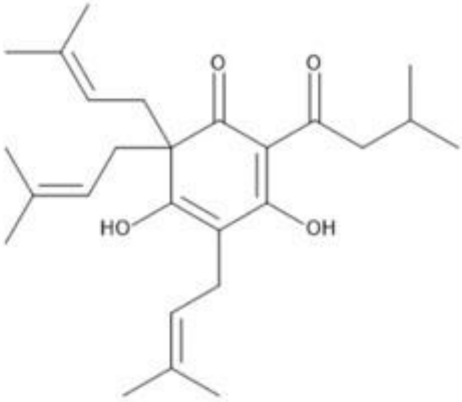	Lupulone
	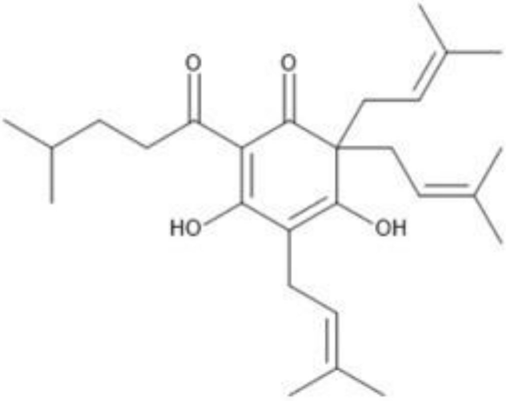	Prelupulone

Hard Resins/ Polyphenols

Catechins

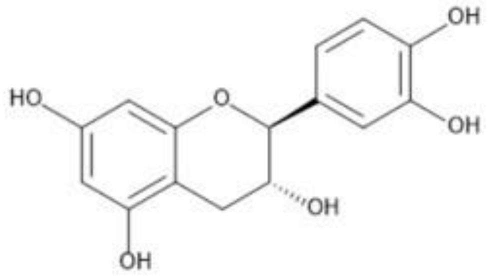

(-)-Catechin

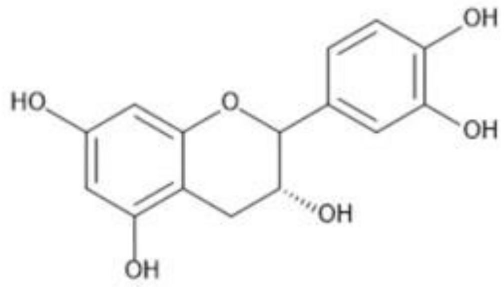

Epicatechin

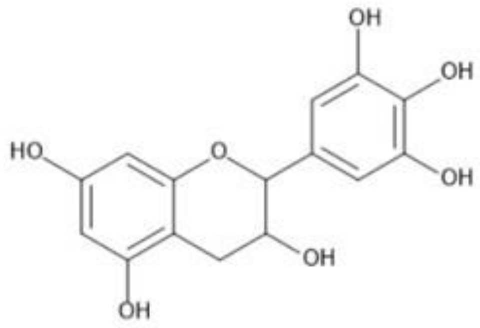

Epigallocatechin

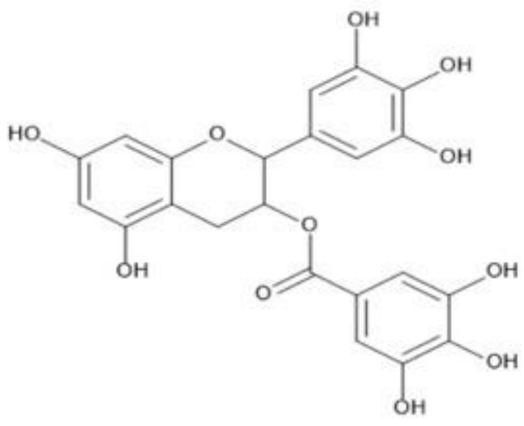

Epigallocatechin-3-O-gallate

**Hard Resins/ Polyphenols**		
Flavonols	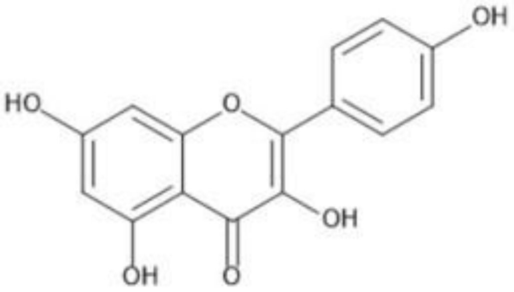	Kaempferol
	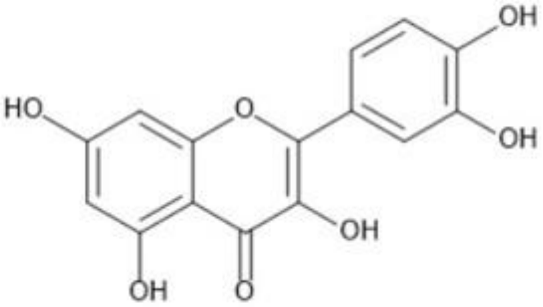	Quercetin
Multifidol glucosides	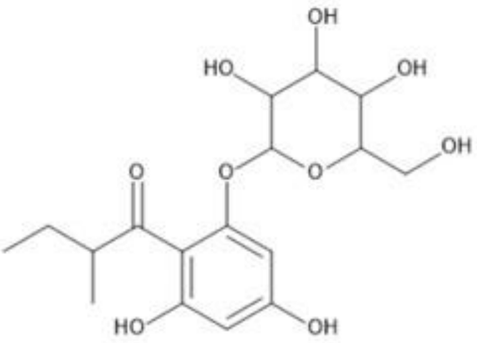	Multifidol glucoside
Hydroxybenzoic acids and derivatives	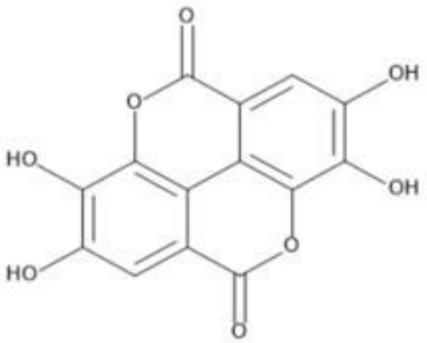	Ellagic acid

**Hard Resins/ Polyphenols**<br><br>Hydroxybenzoic acids and derivatives	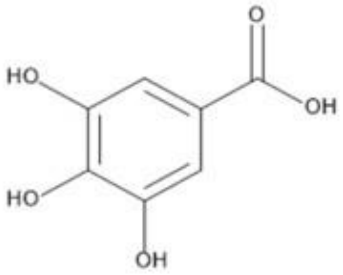	Gallic acid
Hydroxycinnamic acids and derivatives	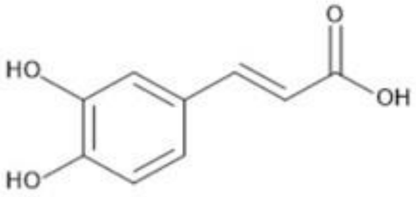	Caffeic acids
	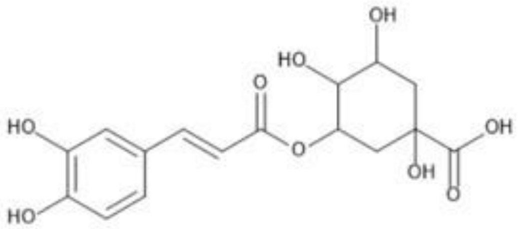	Chlorogenic acids
	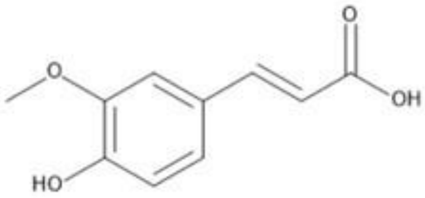	Ferulic acid
	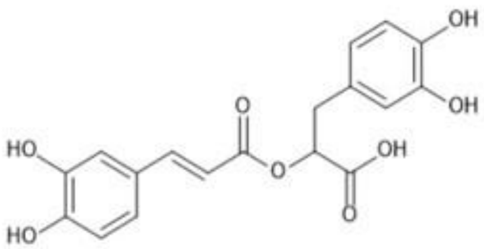	Rosmarinic acid
Prenylflavonoids	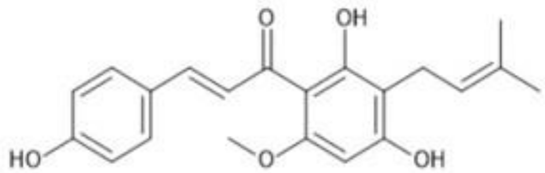	Xanthohumol

**Hard Resins/ Polyphenols**		
Prenylflavonoids	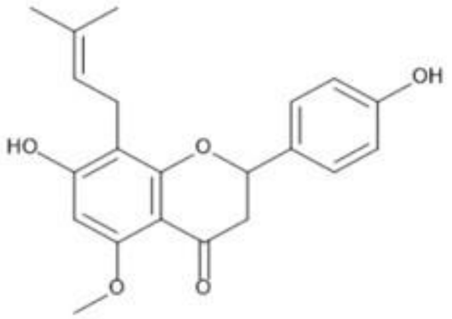	Isoxanthohumol
	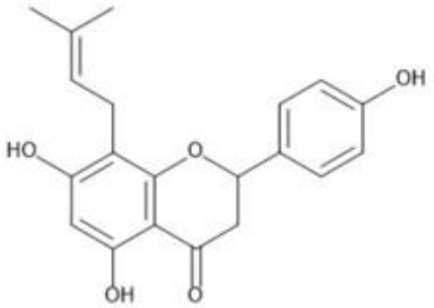	8-prenylnaringerin
Stilbenes	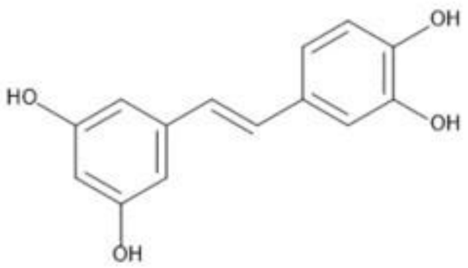	Piceatannol
	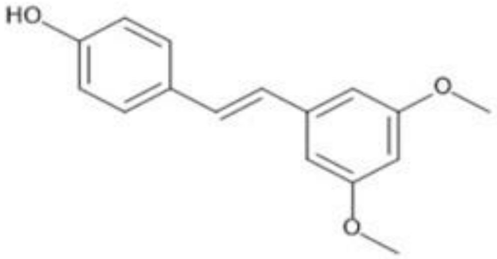	Pterostilbene
	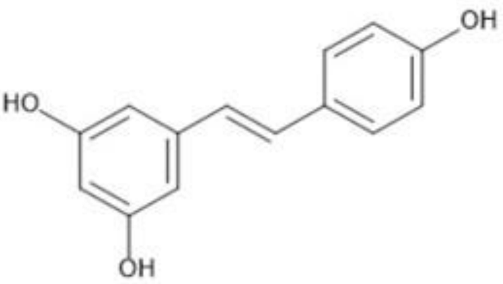	Resveratrol
